# Phospholipids that Plug the Pores of Cholesteryl Ester Transfer Protein Control Its Triglyceride Transfer

**DOI:** 10.34133/csbj.0122

**Published:** 2026-06-01

**Authors:** Sukriti Sacher, Praveen Singh, Abhishek Mukherjee, Akash Kumar Bhaskar, Kausik Chakraborty, Laurent Counillon, Shantanu Sengupta, Mallorie Poet, Arjun Ray

**Affiliations:** ^1^ Department of Computational Biology, Indraprastha Institute of Information Technology, New Delhi 110020, India.; ^2^ Buck Institute for Research on Aging, Novato, CA 94945, USA.; ^3^ Ambinova Technologies Pvt Ltd., New Delhi 110039, India.; ^4^ Division of Pharmaceutical Sciences, St. Jude Children’s Research Hospital, Memphis, TN 38105, USA.; ^5^ Institute of Genomics and Integrative Biology, New Delhi 110025, India.; ^6^ Université Côte d’Azur, CNRS, Laboratoire de PhysioMédecine Moléculaire (LP2M), Laboratories of Excellence Ion Channel Science and Therapeutics, Nice 06107, France.

## Abstract

Cholesteryl ester transfer protein (CETP), a key drug target in cardiovascular disease, primarily regulates plasma circulating levels of lipids (within high, low, and very-low-density lipoproteins). In addition to its terminal openings, CETP has 2 additional openings, plugged by a phospholipid (PL) each. However, the mechanism, by which CETP moves lipids between lipoproteins, and roles of PL plugs in CETP function remain elusive. Further, small-molecule inhibitors targeting CETP tunnel displace PL during CETP inhibition. Here, using steered molecular dynamics simulations followed by *in vitro* mutagenesis, we show that CETP-bound PLs are indispensable in establishing the optimal architecture of CETP tunnel. PLs were critical in synchronizing domain movements of CETP while accelerating triglyceride traversal through the tunnel and their activity regulated through salt bridge interactions. Most notably, PLs bound within the CETP tunnel accelerated lipid movement through a novel “gliding” mechanism, in which a bound PL directly facilitates the movement of a second lipid species. Structural and functional analyses revealed that lipid traversal through the central tunnel of CETP was facilitated through hydrophobic-interaction-mediated diffusion. Further, conserved phenylalanine flaps regulated lipid movement by concerted opening and closing to prevent lipid backflow in the absence of an active motor. This study provides in-depth understanding of the mechanism of lipid exchange by CETP, guided and accentuated by its interaction with PLs. The conserved nature of these critical structural elements suggests that this mechanism may extend to other members of this family, expanding our understanding of lipid transport in this clinically important protein class.

## Graphical Abstract

**Figure:**
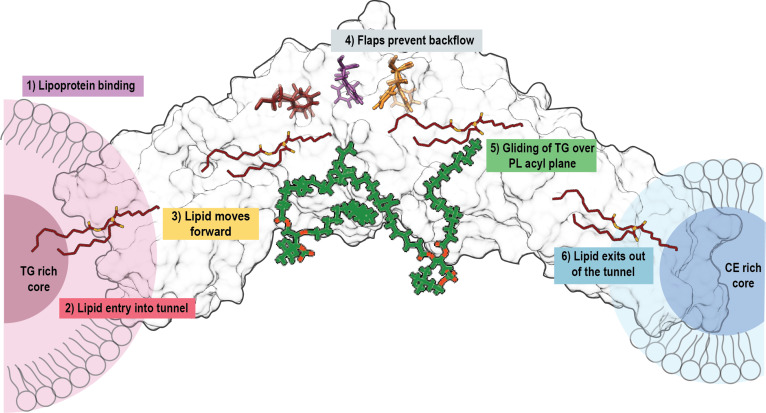

## Introduction

Cholesteryl ester transfer protein (CETP) is a plasma glycoprotein uniquely responsible for bidirectional transfer of cholesteryl esters (CEs) from high-density lipoproteins (HDLs) in exchange for triglycerides (TGs) from low-, intermediate-, and very-low-density lipoproteins [1]. This reciprocal exchange depletes HDL of CEs while enriching it with TGs, accelerating its clearance from circulation [2–4]. CETP is an active target for therapeutic intervention in cardiovascular disease, owing to its proatherogenic role in modulating plasma lipoprotein composition [5–7]. Clinical development has been fraught with setbacks: Torcetrapib (a CETP inhibitor) causes increased mortality owing to off-target toxicity [8]; dalcetrapib and evacetrapib show insufficient efficacy [5,9]; and anacetrapib, despite lowering coronary events, has been withdrawn owing to its prolonged half-life [5,10]. These failures underscore the critical need for elucidating the structure–function relationship of CETP to improve therapeutic interventions.

Two mechanistic paradigms have been proposed for CETP-mediated neutral lipid transfer: a shuttle model, wherein CETP transiently associates with individual lipoproteins [4], and a bridge or tunnel model, in which CETP simultaneously interacts with 2 lipoproteins, enabling direct lipid flow through its elongated hydrophobic tunnel [4]. Electron microscopy supports the formation of both binary and ternary CETP–lipoprotein complexes [11,12]; therefore, resolving the precise molecular mechanism at high resolution is challenging. CETP contains a continuous hydrophobic tunnel made by N- and C-terminal β-barrel domains (Fig. S1D), both of which associate with lipoproteins [11,13–15]. The N-terminal barrel preferentially engages HDL, whereas the C-terminal barrel interacts with TG-rich lipoproteins [11,12]. Lipid entry occurs via the open termini and is governed by concentration gradients across lipoprotein surfaces [16]. The CETP tunnel can accommodate 2 CEs [17], as observed in the crystal structure, or 2 TG molecules [18], as shown using modeling. The tunnel is occluded in the middle by 2 phospholipid (PL) molecules, 1,2-dioleoyl-*sn*-glycero-3-phosphocholine (DOPC), embedded such that their hydrophobic tails are sequestered within the tunnel and their polar headgroups remain solvent exposed (Protein Data Bank [PDB] IDs: 2OBD and 4EWS) [15,17]. Earlier, these PLs have been thought to be exchanged during lipid transport [17]; however, studies with *CETP* transgene into *PLTP* knockout mice have revealed a lack of PL transfer activity by CETP [19], thereby proposing PLs plugging CETP as important structural elements that shield the tunnel from aqueous exposure [19,20]. However, entry of PLs into the CETP tunnel and their structural rearrangement (particularly orientation) in its binding pocket remain inexplicable. In addition, bactericidal permeability increasing (BPI) protein (PDB ID: 1BP1), a structurally homologous protein of CETP, also harbors PLs within its concave groove with an orientation similar to that of CETP [21], implying a conserved architectural role.

CETP activity has been well studied using *in vitro* biochemical assays that quantify mass CE or TG transfer from donor HDL/low-density lipoprotein/liposomes to acceptor particles [16,22,23]. Furthermore, electron microscopic studies have described CETP binding to lipoproteins at a single-particle resolution and aided in the monitoring of changes in lipoprotein size owing to CETP-mediated remodeling [11–13,24]. Because of the limited resolution of standard biophysical or biochemical techniques, molecular dynamics (MD) simulations are the preferred tool for evaluating lipid transport via CETP, as they provide residue-level mechanistic insights. However, existing computational studies have largely focused on CE transport and not TG transport while omitting the structural influence of PLs.

Initial classical and coarse-grained MD simulations of CETP–HDL (binary) complex successfully modeled the entry of CE identifying key residues for anchoring (W105, W106, and W162) and entry facilitation (F35, F93, and F147) [20]. Subsequently, steered MD (SMD) has demonstrated the complete traversal of CE through the CETP tunnel using a minimal ternary complex comprising CETP embedded within lipid monolayers [25]; this study mapped the entire translocation pathway, identifying the N-barrel entry points (F115, R158, and F167), neck-region energy barriers (I15, L23, A202, I205, L206, F263, F265, and M43), and the C-barrel exit residues (F301 and M412). However, this ternary model excluded the bound PLs, overlooking a critical steric and biochemical constraint that likely influences transport mechanism. The mechanism of CETP inhibition by small molecules (torcetrapib and anacetrapib) has been elucidated by calculating the free energy changes during CE movement [26]. These studies suggest that CETP inhibitors physically occlude the tunnel, forcing CE to take alternative exit paths [26]. While it is known that these inhibitors displace N-PL (PL between the N-barrel and neck) upon binding [15], the specific impact of PL displacement in overall lipid dynamics remains unresolved. To date, mechanistic studies on the role of PLs are limited to comparing apo-CETP to CETP-bound PLs, suggesting that PLs influence domain flexibility and promote CE linearization [27,28].

**Fig. 1.**
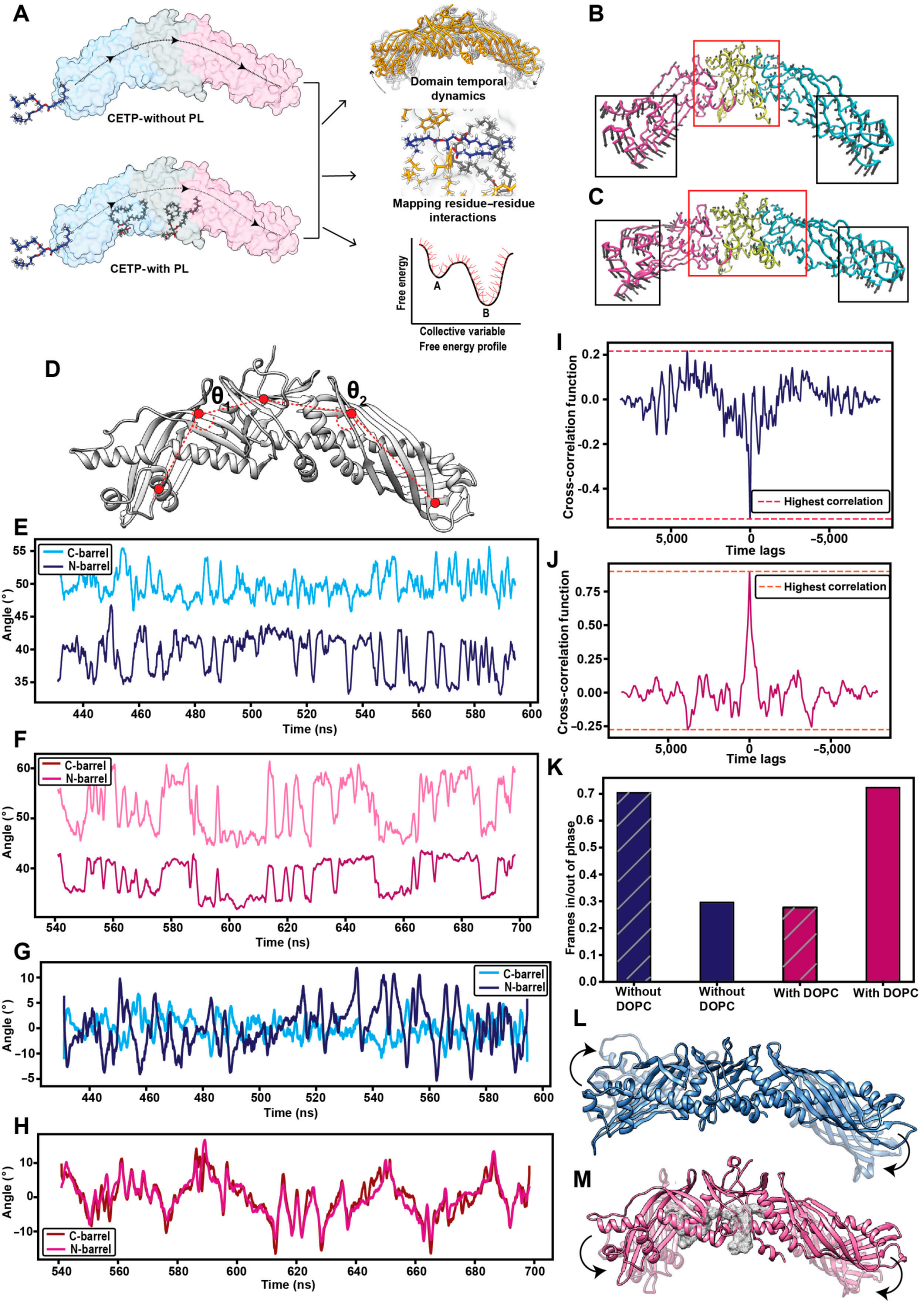
Phospholipid (PL) plugs result in a switch in synchronicity of oscillations of cholesteryl ester transfer protein (CETP) barrels. (A) Schematic of the study design. Normal model analysis depicting the 2 dominant modes of motion in (B) absence and (C) presence of PL plugs. The length of the arrows depicts amplitude, and the arrowheads depict the direction of motion. The protein is colored according to its 3 domains: C-barrel (pink), neck (yellow), and N-barrel (blue). (D) Vectors used to compute barrel movement. Dynamic movements of the C-barrel and N-barrel domains of CETP in the (E) absence and (F) presence of PL plugs. Data for the last 150 ns poststabilization are shown. Rate of change of phase angle of the time series representing the movement of C-barrel and N-barrel in (G) absence and (H) presence of PL plugs. Cross-correlation between movements of the C-barrel and N-barrel in the (I) absence and (J) presence of PL plugs. (K) Duration of simulation, in which the 2 barrels remain synchronous (in-phase) or asynchronous (out-phase) in the presence or absence of PL plugs, respectively. The duration is depicted as a ratio of the number of frames in/out phase across triplicate simulations to the total number of frames. Schematic representation of (L) asynchronous and (M) synchronous movement of the CETP barrels.

Despite these insights, substantial gaps remain in our understanding of lipid transfer by CETP. First, it is unclear whether TGs follow the same translocation mechanism as CEs or whether their larger molecular volume necessitates a distinct transport mechanism. Second, the functional role of embedded PLs remains debated: Are they merely passive structural components that reduce aqueous exposure of the CETP tunnel, or do they actively modulate the energetic landscape of lipid transit? Last, how discrete events such as lipid entry, traversal, and exit are integrated into a unified mechanistic model of lipid exchange remains to be determined. To address these gaps, we performed extensive classical and SMD simulations of CETP in both PL-bound and apo states to elucidate the mechanism of TG transport (Fig. 1A). By identifying the critical residues governing TG movement and PL-mediated activity, this study provides a unified mechanistic foundation for understanding lipid transfer within CETP.

## Materials and Methods

### MD simulation setup

The system comprising CETP with PLs (PDB ID: 2OBD) contained 2 DOPCs in the crystal structure orientation or CETP without PLs was represented by CHARMM36 [29,30] and TIP3P water model [31]. The choice of phosphatidylcholine was supported by mass spectrometry, which showed that phosphatidylcholine species (predominantly with oleoyl and palmitoyl chains) are the specific occupants of the binding pocket [17]. The simulation protocol included energy minimization of the system using the steepest descent algorithm, followed by production runs in the NPT (isothermal–isobaric) ensemble using a modified Berendsen thermostat [32] at 300 K and Berendsen barostat [33] using 1 bar as the reference pressure and a time constant of 2.0 ps with a compressibility of 4.5 × 10^−5^ bar using the isotropic scaling scheme. Periodic boundary conditions were used, and long-range electrostatic interactions were treated with the particle mesh Ewald method [34] using 0.12-nm grid spacing combined with a fourth-order B-spline interpolation for calculating the potential and forces in-between grid points. The real-space cutoff distance and van der Waals cutoff were set to 1.2 nm. The bond lengths were fixed using the LINCS algorithm [35], and a time step of 2 fs for numerical integration of the equations of motion was used. Three independent trajectories, each of 650 ns at 300 K, were carried out for CETP with PLs (CETP-with DOPC). For CETP without PL (CETP-without DOPC), simulations were carried out for 600 ns at 300 K. Simulations for CETP mutants R201A and R424A were carried out for 700 ns at 300 K. All simulations were carried out using GROMACS v2020.1 [36–38] (Table 1).

**Table 1.** Summary of the simulations performed in this study

Method	System	System size	Number of replicates	Time (ns)
**Classical MD**	CETP-with DOPC	219,423	3	650
	CETP-without DOPC	219,315	3	600
	R201A-CETP	258,425	1	700
	R424A-CETP	258,437	1	700
**Steered MD**	CETP-with DOPC	214,826	6	81
	CETP-with DOPC (N-barrel to C-barrel; reverse pull)	214,883	3	43.5
	CETP-without DOPC	214,877	6	75
	R201A-DOPC	214,807	3	40.5
	R424A-DOPC	214,801	3	37.5
**Umbrella sampling**	CETP-with DOPC	207,009	3	50 ns each, ~7.2 μs
	CETP-without DOPC	214,877	3	50 to 100 ns each, ~ 9.9 μs

MD, molecular dynamics; CETP, cholesteryl ester transfer protein; DOPC, 1,2-dioleoyl-*sn*-glycero-3-phosphocholine.

### SMD simulation setup

Following the conventional MD run, the last frame of the trajectory was used as the starting point for SMD simulations. One TG (10:0/10:0/10:0), chosen on the basis of computational tractability, represented by modified CHARMM36 [39] was manually placed at the opening of the C-barrel of CETP [11]. SMD simulations were performed independently on each of the 2 systems in the absence or presence of PL plugs to elucidate the path of TG transfer. TG was pulled through the CETP tunnel using constant-velocity SMD simulations. The pulling vector was defined on the basis of the center of mass of the steered group, i.e., the tail region of TG and center of mass of CETP tunnel residues in the direction of the N-barrel. Considering the asymmetric shape of CETP, its center of mass was dynamically switched from the C-barrel to the neck and finally the N-barrel, depending on the location of the steered group. Therefore, entire pull simulation through the CETP tunnel was executed in 4 phases. Constant-velocity simulations were performed by implementing a force constant of 1,000 kJ/mol per square nanometer. For each system, either with or without PL, TG was pulled with a velocity of 1 nm/ns in 6 replicates. For the CETP mutants (R201A and R424A), SMD simulations were performed in triplicates (Table).

### Calculation of the potential of mean force

Umbrella sampling simulations were performed on 3 different paths taken by TG in the presence or absence of PL plugs, derived from 3 SMD simulations. Starting from the initial position of TG at the mouth of the C-barrel, the path traversed through the tunnel was sampled at every 0.1 nm. A force constant of 500 kJ/mol per square nanometer was used. To ensure sufficient overlap of histograms along the RC (reaction coordinate), each window was thoroughly equilibrated for 1 ns and subjected to a production run for 50 ns. The histograms obtained were unbiased and combined to produce the potential of mean force (PMF) using the weighted histogram analysis method by excluding the first 20 and 50 ns from the trajectory in the presence and absence of PL plugs, respectively [40]. An average PMF profile was generated by considering the mean potential at each point along the reaction coordinate with bootstrapping (100 runs) to generate errors. The first and last bins of the reaction coordinate were assumed to be neighbors for generating a periodic PMF. To assess convergence, the stability of the PMF profiles was monitored over time. Specifically, the PMFs computed using progressively longer trajectory segments were computed (data accumulated from 10 to 20 ns, 10 to 30 ns, 10 to 40 ns, and 1 to 50 ns of each window). The resulting profiles showed negligible differences, indicating that the free-energy landscapes had converged well before the end of the 50-ns sampling period (Table 1).

### Plasmids and mutants

Wild-type (WT)-*CETP* plasmid was a kind gift from T. B. Strøm, Department of Medical Genetics, Oslo University Hospital, Norway [41]. Using pcDNA3.1-WT-CETP-V5 as a template, 5 mutants were generated using the QuikChange II Site-Directed Mutagenesis Kit (Agilent Technologies, Santa Clara, CA, USA) according to the manufacturer’s instructions. Plasmids were isolated using the Plasmid Midi Kit (Macherey-Nagel GmbH & Co. KG, Düren, Germany). Plasmid integrity was verified by Sanger sequencing. The sequences of the oligonucleotides used are provided in (Table S1).

### Cell culture and transfection

Human embryonic kidney (HEK) 293-F cells (6 × 10^5^ cells per well) were seeded in a 6-well plate (Nunc, Villebon-sur-Yvette, France) and cultured in Dulbecco’s modified Eagle’s medium (Gibco, Villebon-sur-Yvette, France) supplemented with 10% fetal bovine serum (Sigma-Aldrich Chimie, Saint-Quentin-Fallavier, France) and 1% penicillin–streptomycin antibiotic (Gibco, Villebon-sur-Yvette, France). Cells at 50% confluence were transfected with 1 μg of DNA using a jetPRIME transfection kit (Sartorius Polyplus SAS, Illkirch-Graffenstaden, France). After 2 h, cells were washed with phosphate-buffered saline (Sigma-Aldrich Chimie, Saint-Quentin-Fallavier, France) and were incubated in Freestyle 293 expression media (Gibco, Villebon-sur-Yvette, France). Cells were harvested 48 h posttransfection and were lysed using radioimmunoprecipitation assay lysis buffer (150 mM NaCl, 1% d’IGEPAL CA-630, 0.5% sodium deoxycholate, 0.1% sodium dodecyl sulfate, and 50 mM tris [pH 8]) containing a protease inhibitor cocktail (protease and phosphate inhibitor, Sigma-Aldrich Chimie, Saint-Quentin-Fallavier, France). The spent medium was centrifuged at 1,200 rpm for 5 min to remove cell debris. Protein concentrations of the lysates and supernatants were determined using RC DC Protein Assay (Bio-Rad Laboratories Inc., France).

### Western blotting

Equal amounts (30 μg) of protein samples were separated by 10% sodium dodecyl sulfate–polyacrylamide gel electrophoresis. Prestained Opti-Protein Marker (Applied Biological Materials Inc., Richmond, BC, Canada) was used as a molecular weight marker. Separated proteins were blotted on a polyvinyl difluoride membrane (Amersham Hybond, 0.2 mm; Sigma-Aldrich Chimie, Saint-Quentin-Fallavier, France). The membrane was blocked using skimmed milk for 1 h at 25 °C. Subsequently, the membrane was incubated with an anti-V5 antibody (Invitrogen, Villebon-sur-Yvette, France, R96025) (1:1,500 for supernatant and 1:5,000 for lysate) for detecting V5-tagged CETP or anti-β-actin antibody (Sigma-Aldrich Chimie, Saint-Quentin-Fallavier, France A5441), which was used as an internal control for cell lysate. An anti-mouse immunoglobulin G (IgG) conjugated with horseradish peroxidase (goat anti-mouse IgG[H+L] secondary antibody horseradish peroxidase [HRP], #32430, Thermo Fisher Scientific, Villebon-sur-Yvette, France] was used as a secondary antibody. Immunoreactive protein bands were detected using (Immobilon Western Chemiluminescent HRP Substrate, Millipore) were captured on Fusion Fx (Vilber Lourmat, Marne-la-Vallée, France). CETP expression was analyzed using ImageJ v.1.54p and normalized with respect to β-actin expression.

### CETP activity assay

Lipid transfer activity of CETP was measured using 4 μg of protein from each supernatant (WT or mutant), using the CETP Activity Assay Kit (MAK106, Sigma-Aldrich Chimie, Saint-Quentin-Fallavier, France) according to the manufacturer’s instructions. Briefly, fluorescently labeled donor particles containing a CE analog were incubated with the supernatant and acceptor particles in a sealed black flat-bottomed 96-well plate (view plate 96 black, clear bottom; Revity, France) for 3 h at 37 °C in a water bath. Fluorescence intensities were measured using excitation and emission wavelengths of 465 and 535 nm, respectively, on a spectrofluorometer (SAFAS Xenius, SAFAS, Monaco) at 400 V.

### Statistical analysis

All experiments were repeated at least thrice. The data are presented as means ± standard deviation. A Student’s *t* test was used to compare between 2 groups. The Mann–Whitney *U* test was used to compare the volume and hydrophobicity profiles between CETP-with DOPC and CETP-without DOPC in MD simulations. Statistical significance was set at *P* < 0.05.

## Results

The structure of CETP (PDB ID: 2OBD) was simulated with and without its DOPC plugs in triplicates for deciphering the role of PL plugs in lipid transfer. The bound PL simulations assumed the PL orientation observed in crystal structure (PDB IDs: 2OBD, 4EWS, and 1BP1) to ensure physiological relevance despite unclear in vivo acquisition. During unrestrained simulation, the root mean square deviations plateaued at 3 to 5 and 2 to 3 Å for CETP simulations with or without DOPC, respectively (Fig. S1A and B). Structures sampled poststabilization showed a significant increment in tunnel volume within the neck and adjacent C-barrel region of CETP in presence of PLs, while tunnel hydrophobicity remained unchanged in both systems (Fig. S1C to E).

### PL plugs synchronize the movement of CETP barrels

In the crystal structure, the 2 barrels of CETP appeared bent, providing it a boomerang shape [17]. However, the average tilt of the CETP barrels postsimulation differed drastically (47.5° and 45° in the absence and presence of PLs, respectively) from that of the crystal structure (72.9°) (Fig. S2A and B). Normal mode analysis indicated that the terminal regions of the barrel exhibited large movements toward each other irrespective of PL plug status of CETP (Fig. 1B and C). However, in the presence of PLs, the neck and its adjacent regions, in addition to the barrel ends, exhibited small movements (Fig. 1C).

The differential motion of CETP in the presence and absence of PLs intrigued us to investigate the rhythmicity within barrel motions. We calculated the angle formed by the N-barrel and C-barrel with respect to the neck across simulation time (Fig. 1D). The N-barrel oscillated between 35° and 40°, irrespective of the PL status (Fig. 1E and F). In contrast, the angle of C-barrel oscillations showed mean fluctuations of approximately 10° (between 47° and 57°) in the presence of PLs compared to 2° (between 50° and 52°) in the absence of PLs (Fig. 1E and F). Further, the movements of the N-barrel and C-barrel negatively correlated in the absence of PLs (Fig. 1I), whereas they positively correlated when the PL plugs were present (cross-correlation coefficient, 0.8 at 0 lags) (Fig. 1J). This implies that the movements of the N-barrel and C-barrel in CETP-with DOPC are temporally similar and movements of the 2 barrels are synchronized with each other. To determine the phase relationships between barrel oscillations, we performed a Hilbert transformation [*x*^**^**^(*t*)] of the angle–time series [*x*(*t*)]. On overlaying *x*^**^**^(*t*) of the 2 barrels, the rates of their phase change varied in the absence of PLs (Fig. 1G). In contrast, the 2 barrels exhibited synchronous changes in phase in the presence of PL plugs (Fig. 1H). The number of frames exhibiting such concerted movement was much higher (>70% of simulation time) in the presence of PLs than in their absence (Fig. 1K), implying that the movement of the 2 barrels was synchronized in the presence of PL plugs (Fig. 1M) than in their absence (Fig. 1L).

### PL plugs alter the route taken by TG ensuring successful traversal through the tunnel

**Fig. 2.**
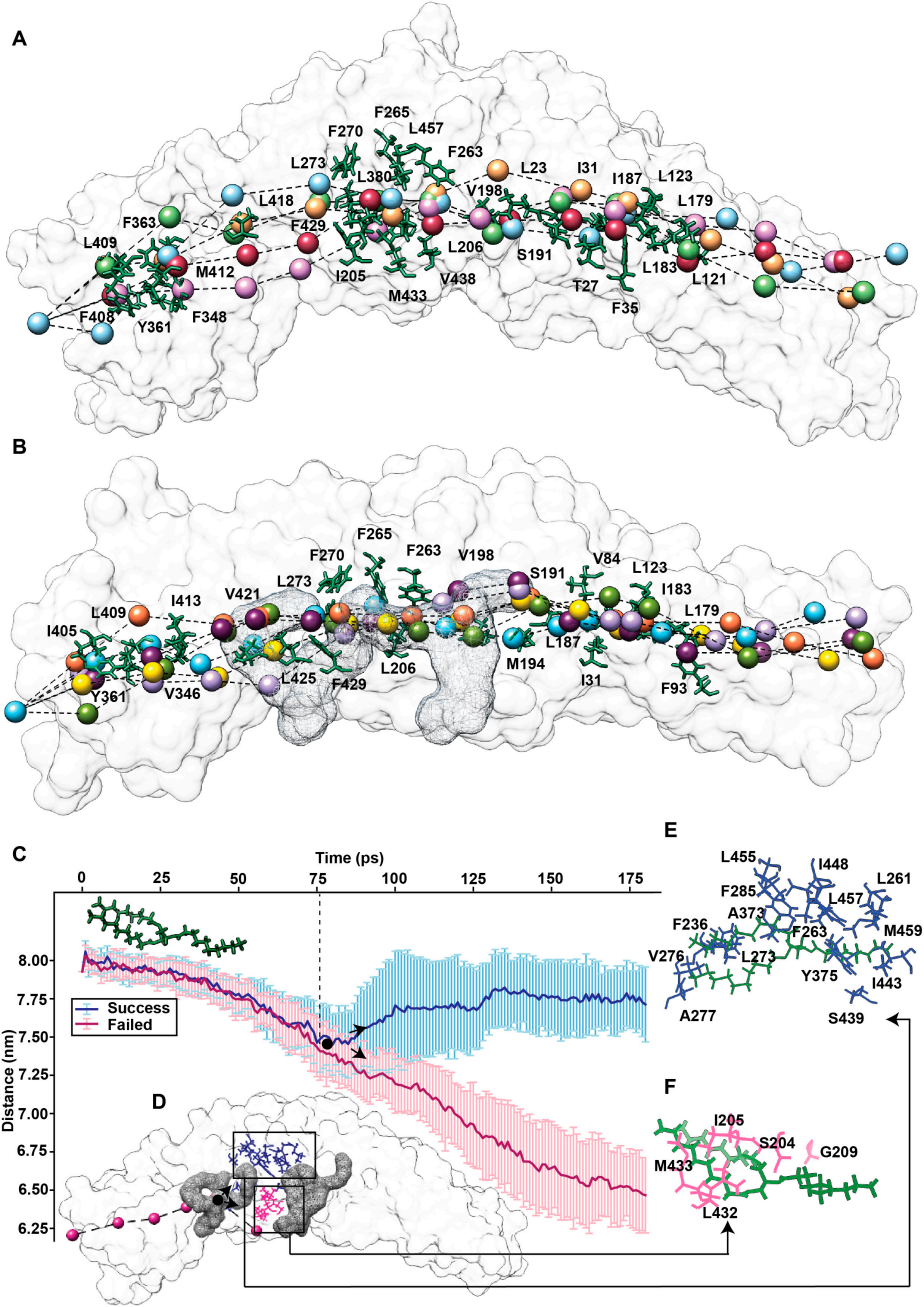
Triglyceride (TG) transfer through cholesteryl ester transfer protein (CETP). The center of mass of the glycerol moiety of TG was traced as it moved through the tunnel in each replicate simulation of CETP (A) without and (B) with phospholipids (PLs), determining the path taken by TG through the CETP tunnel. The residues within a radius of 0.3 to 0.6 nm that stay in contact TG for >1 ns of simulation time in 6 replicates are highlighted. Multiple paths derived from several independent replicate simulations are depicted. (C) The average path taken by TG (across 43 replicate simulations) either directs it through the neck (blue) or leads it out into the milieu through the space in the floor of CETP (pink). Error bars depict standard deviations. (D) The inset shows the path taken by TG, which leads to its fall out, and location of PL plugs on CETP. The inflection point is depicted as a black dot. Residue clusters located toward the (E) roof and (F) floor of the protein toward which TG gravitates.

To study the TG transfer dynamics through CETP tunnel, we placed a TG molecule at the mouth of the C-barrel and performed SMD on these 2 systems in the absence or presence of PLs [11,12] (Fig. S2C). A pull was considered successful if TG entered from the C-barrel and exited through the N-barrel end after traversing the entire tunnel (Table S2 and Movie S1). Next, we identified all the hydrophobic clusters lining the tunnel that TG encounters during transit (Fig. 2A and B and Tables S3 and S4). The paths taken by TG clustered together in the presence of PLs, whereas they were scattered (specifically in the 2-barrel regions) when PLs were absent (Fig. 2A and B). A comparison between the average path taken by TG and the previously identified path for CE [25,26] indicated that it interacted with 18 common residues (I15, L23, F35, F93, V198, I205, L206, F263, F265, F270, Y361, F363, M412, F408, F429, L425, F429, and M433; Fig. S2D and Table S5).

In the absence of PLs, although TG entered the tunnel via the C-barrel end (7 instances), it exited downward through the space between the C-barrel and neck in 2 of the replicates (Movie S2 and Table S6). We revisited these failed simulations for elucidating the cause of this premature exit. Starting with a simulation frame such that TG had reached the end of C-barrel, TG was pulled toward the N-barrel with random velocities. In 51% of these simulations (43 replicates), TG dropped out through the space between the barrel and neck when PL plugs were absent (Fig. 2C and Table S6). However, not a single fall was observed in the presence of PLs. Next, we calculated the residues that made contact with TG and identified 2 hydrophobic patches toward which TG gravitated. The first patch was located toward the roof of the neck region and composed of residues F236, L261, F263, F265, L273, V276, A277, A373, Y375, S439, I443, I448, L457, M459, and L485 (Fig. 2E). The second patch was located toward the floor of the neck region and composed of residues S204, I205, G209, L432, and M433 (Fig. 2F). Therefore, TG navigated through the tunnel by moving from one hydrophobic cluster to another. A bifurcating inflection point lies at the interface of the C-barrel and neck, beyond which 2 fates for TG exist: TG either reaches the center of the neck or falls out through the opening in the floor of the protein (Fig. 2D). Interestingly, the first PL plug was located at this inflection point, suggesting that the PLs physically seal CETP such that TG does not slip out during transit. Moreover, favorable interactions with the acyl tails reroute TG, such that it always moves toward the hydrophobic patch at the roof of CETP, reducing the probability of fallouts.

### Side-chain motion of the residues lining the neck region allows the passage of TG into the tunnel

**Fig. 3.**
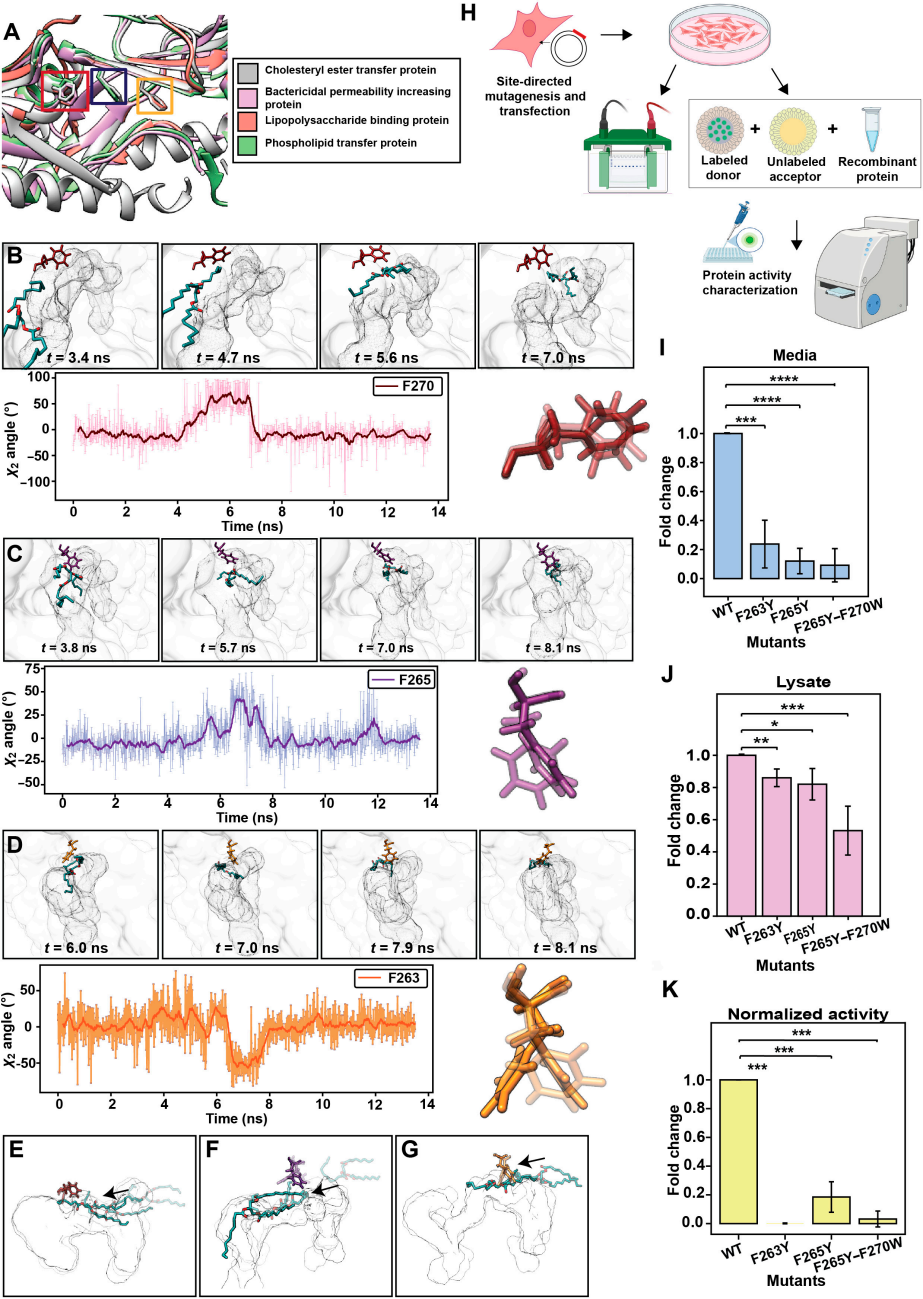
Movement of the flap residues lining the neck region of cholesteryl ester transfer protein (CETP). (A) Location of flaps in other members of the bactericidal permeability increasing (BPI)/lipopolysaccharide binding protein (LBP) family. Protein Data Bank (PDB) structures of CETP (PDB ID: 2OBD), BPI (PDBID: 1EWF), LBP (AF-P18428-F1), and phospholipid transfer protein (PLTP) (AF-P55058-F1) overlaid using Chimera. F270, F265, and F263 are highlighted in red, blue, and yellow, respectively. Changes in normalized *χ*_2_ angle of (B) F270, (C) F265, and (D) F263 throughout simulation. Snapshots of the simulation depict the movement of Phe side chains as triglyceride (TG) approaches it. The solid line depicts the moving average across 20 ps in a simulation. The line plot represents the changes in *χ*_2_ angle in real time. Snapshots depicting the movement of (E) F270, (F) F265, and (G) F263 when TG was pulled from N-barrel to C-barrel. (H) Schematic depicting the study design for experimental validation. Bar plot depicting fold changes in CETP expression of the mutants with respect to that of the wild-type (WT) (I) media and (J) cell lysate. (K) Measurement of CETP activity normalized with respect to protein expression. Values from 3 biological replicates are shown. The data are presented as means ± standard deviation. *P < 0.05, **P < 0.01, ***P < 0.001, and ****P < 0.0001; ns, not significant (*t* test).

Of the residues that made a contact with TG for an extended duration, 3 Phe residues were uniquely oriented and also extensively interacted with PL acyl tails. These residues (F270, F265, and F263) were located on the roof of CETP and had their side chains buried inside the cavity. Interestingly, these Phe residues are conserved in other members of the BPI family; F265 is found in PLTP (PL transfer protein); F263 is found in lipopolysaccharide binding protein (LBP); and F270 is present in PLTP, LBP, and BPI in addition to CETP (Fig. 3A).

To ascertain whether these residues altered the route of TG, we calculated the torsion angles of their *χ*_2_ side chains throughout the pulling simulations. These residues underwent frequent rotamer switching irrespective of the PL status of CETP (Fig. S3A and B). However, in the presence of PL plugs, as TG slid past these residues, they rotated by approximately 50° to pave the way for TG movement (Fig. 3B to D). Once TG passed, the side chains went back to their original orientation. This motion of the 3 Phe side chains was coordinated, moving sequentially as TG approached them. To test the direction of movement of Phe side chains, we pulled TG in the reverse direction (from the N-barrel to the C-barrel) and observed that the Phe side chains swung open again, allowing TG to pass through (Fig. 3E to G and Fig. S3C to E).

Given the evolutionary conservation and bottleneck location of the Phe flaps, we mutated them to Tyr (F263Y, F265Y, and F265Y–F270Y). HEK293 cells were transfected with plasmids expressing WT-CETP or mutants, and CETP expression in the media and cell lysate were analyzed using Western blotting (Fig. 3H and Fig. S3F and G). All the 3 Phe mutants showed poor CETP expression in the media, with F263Y, F265Y, and F265Y–F270Y expression being 0.25-, 0.20-, and 0.1-fold, respectively, compared to that of the WT (Fig. 3I). For F263Y and F265Y, CETP expression in cell lysate declined by 20%, while the F265Y–F270Y double mutant showed a 50% decline in CETP expression in cell lysate, compared to that of the WT (Fig. 3J). These results clearly indicated that the flap (F263Y, F265Y, and F265Y–F270Y) mutants altered CETP structure, affecting either its folding or secretion, thereby leading to decreased expression. Furthermore, the flap mutants presented highly negative effects on CETP activity, with F263Y showing negligible activity and F265Y and F265Y–F270Y showing >70% decline in activities (Fig. 3K).

### PL plugs facilitate TG movement through a forward sliding motion

**Fig. 4.**
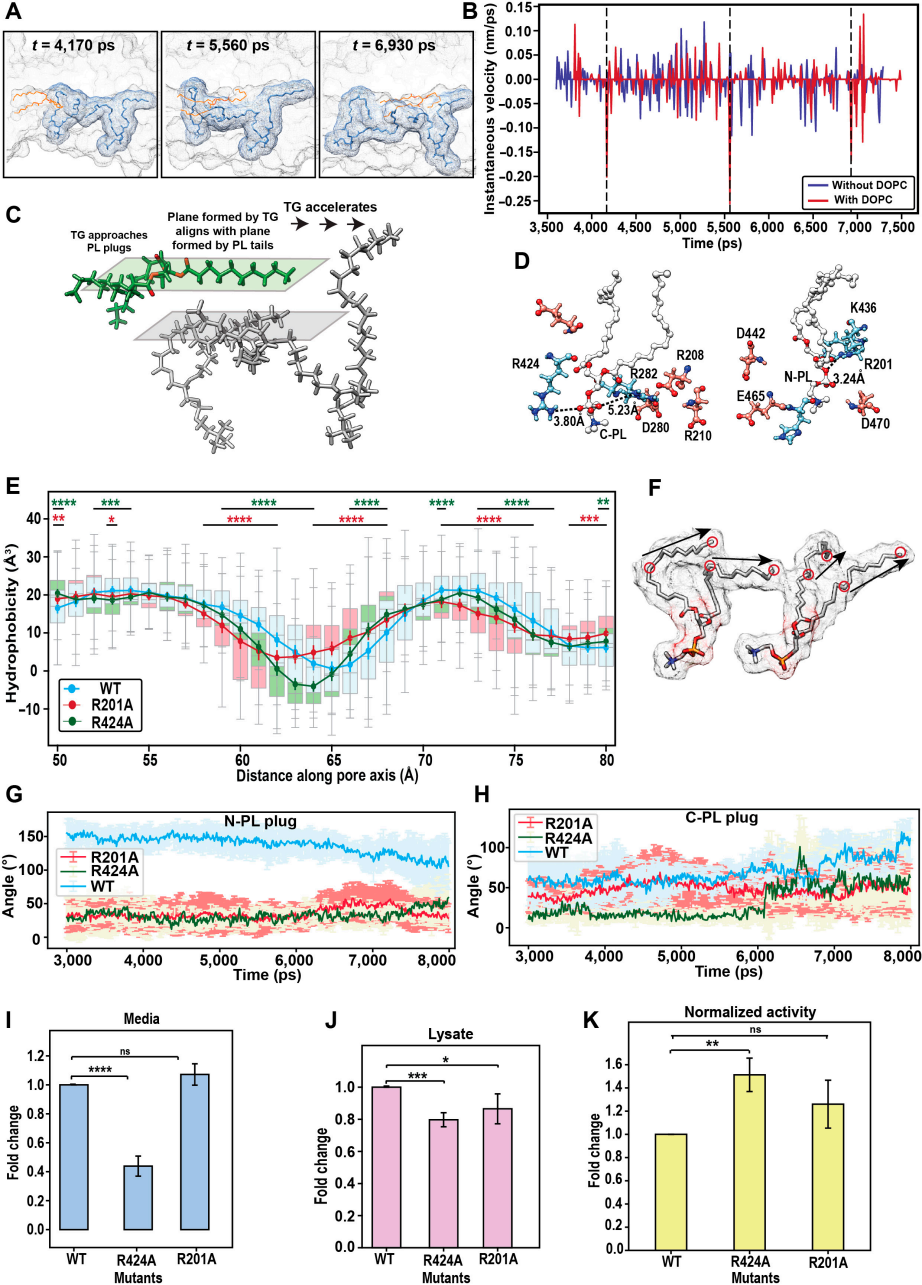
Gliding movement of triglyceride (TG) over phospholipid (PL) acyl tails. (A) The time points, at which a forward gliding movement of TG is observed, depicted through simulation snapshots. (B) Instantaneous velocity of TG through the neck region of cholesteryl ester transfer protein (CETP). (C) Schematic depicting the gliding of 2 hydrophobic planes formed by TG and PL acyl tails. (D) Salt bridge forming residues around C-PL (PL between the C-barrel and neck) and N-PL (PL between the N-barrel and neck) plugs. (E) Tunnel hydrophobicity of the neck region of wild-type (WT) and mutant CETP. The box plot depicts the distribution of hydrophobicity values calculated over 100 frames (poststabilization). Solid lines depict the median value of distribution for the distance along the tunnel axis. The Mann–Whitney *U* test was used for statistical significance of the difference between the profiles of WT and R201A (red) and between those of WT and R424A (green). (F) Schematic of vectors defining the movement of PL acyl tails for (G) and (H). Angle between the acyl tails of (G) N-PL and (H) C-PL through steered molecular dynamics simulations. Bar plot depicting fold changes in CETP expression of the mutants with respect to that of the WT in (I) media and (J) cell lysate. (K) Measurement of CETP activity normalized with respect to protein expression. Values from 3 biological replicates are shown. The data are presented as means ± standard deviation. *P < 0.05, **P < 0.01, ***P < 0.001, and ****P < 0.0001; ns, not significant (*t* test).

As TG moved through the neck region, past the PL tails, it exhibited a forward thrusting movement (Movie S3). This movement was only apparent in the neck region and not during TG entry or exit through the barrels (Fig. 4A). To quantify this thrust, we determined the instantaneous velocity of TG, which increased as TG moved past PL acyl tails (Fig. 4B and Fig. S4B and C). The occurrence of spikes in the instantaneous velocity coincides with an “assisted gliding” motion. Closer observation reveals the formation of a spatial hydrophobic planar surface due to the interaction between PL tails and TG, allowing it to glide forward (Fig. 4C).

To test the validity of the hydrophobic plane’s role in assisting the activity of the protein, we identified the residues in the central pores of CETP that surround PL headgroups. These primarily composed of charged residues (Arg, Lys, Asp, and Glu) that formed salt bridges with the PL headgroups (Fig. 4D and Fig. S5). Removal of the charged Arg side chain was predicted to reduce electrostatic constraint on the PL headgroup, decreasing PL fluctuations and rigidifying the hydrophobic plane formed by the acyl tails. Therefore, we mutated R424 and R201 near C-PL (PL between the C-barrel and neck) and N-PL, respectively, to Ala. *In silico* characterization of the 2 mutants showed increases in tunnel hydrophobicity, specifically in the neck region (Fig. 4E and Fig. S4A). Moreover, the overall fluctuations of PL plugs were reduced in the mutants in comparison to WT CETP (Fig. S4D to G). However, the extremities of acyl tails exhibited a unique orientation, such that they laid parallel to each other (Fig. 4F to H) (angled between the 2 acyl chain extremities of PL was ≤50°) forming a rigid but stable hydrophobic plane (Fig. 4C).

These results were further validated by mutating these residues *in vitro.* HEK293 cells transfected with plasmids expressing WT or mutant CETP were analyzed using Western blotting. R201A showed an expression similar to that of the WT in the media. However, R424A expression was significantly reduced (0.4-fold compared to that of the WT in the media) (Fig. 4I). CETP expression in cell lysate declined by 20% for both R201A and R424A, compared to that of WT CETP (Fig. 4J). Furthermore, both R424A and R201A had significantly higher activities than that of WT CETP (Fig. 4K). The increased stability of the hydrophobic plane may lead to an increase in sliding events contributing to fast lipid transfer through the tunnel in these mutants.

### Mechanism of lipid transfer through CETP

**Fig. 5.**
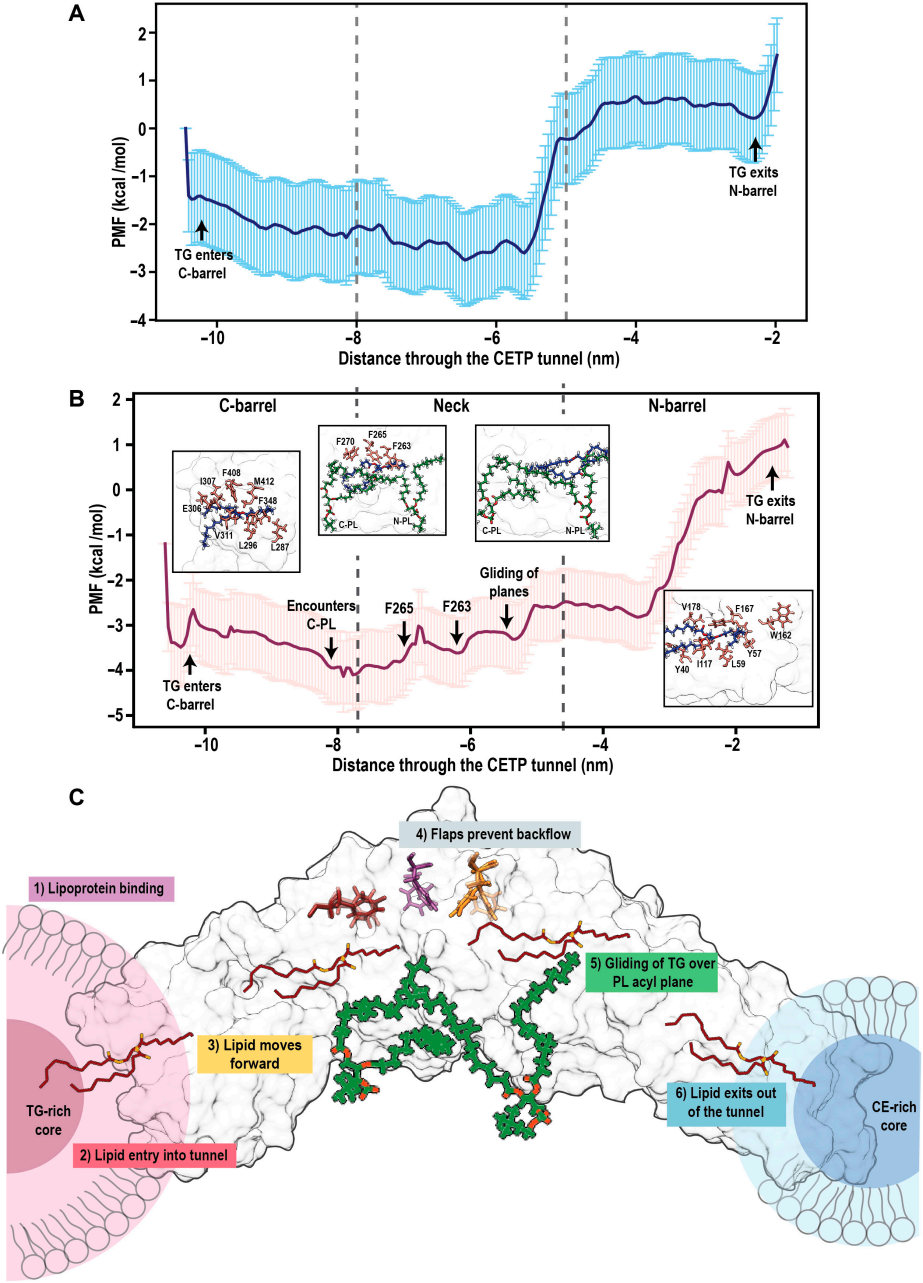
Mechanism of lipid transfer through cholesteryl ester transfer protein (CETP). Potential of mean force profile for the movement of triglyceride (TG) through the CETP tunnel in the (A) absence and (B) presence of phospholipids (PLs). Errors bars depict standard deviations from the mean and are calculated using bootstrapping. The major events that occur in the CETP tunnel are depicted with arrows and snapshots, respectively. (C) Schematic describing the 6 steps involved in the mechanism of TG transfer. PL-induced domain synchrony results in binding of CETP to lipoproteins (step 1). Upon CETP attachment and penetration into lipoprotein surface, neutral lipids present near the surface enter the CETP tunnel owing to a difference in concentration gradients (step 2). Once inside the tunnel, peristaltic forces generated by domain movements of CETP guide the lipid further into the tunnel. In addition, transient contacts with the hydrophobic clusters located inside the tunnel steer the lipid forward (step 3). As an internal check in the neck region, the concerted movement of the flaps ensures no lipid reflux (step 4). Subsequently, the hydrophobic interactions between PL acyl tails and lipid lead to acceleration of lipid forward (step 5). On reaching the end of the tunnel, lipid exits owing to a difference in concentration gradients (step 6).

Next, to deduce the free energy of TG traversal within the CETP tunnel, we calculated the PMF by choosing windows (spaced 0.1 nm apart) along the RC defined by the path taken by TG within the tunnel (Fig. 5A and B). The PMF profile suggested that the entry of TG into CETP tunnel was thermodynamically favorable, characterized by a downhill slope as TG moved from an aqueous to a hydrophobic environment. Notably, this slope was substantially steeper (approximately −3.5 kcal/mol·nm) in the presence of PLs than in their absence (approximately −1.5 kcal/mol·nm) (Fig. 5A and B).

Upon entering the tunnel, TG encountered a cluster of mostly hydrophobic residues (L287, L296, E306, I307, V311, F348, F408, and M412) that formed a shallow barrier of approximately +1 kcal/mol (Fig. 5B). Once past this initial resistance, movement deeper into the C-barrel became stabilizing (gradual descent of −1 kcal/mol). At the interface of the C-barrel and neck (reaction coordinate, −8.1 nm), a shallow well with a depth of 1 kcal/mol was observed, in which the movement was guided by interactions with the acyl tails of PL.

As TG entered the neck, it underwent a 3-step gradual ascent (slope = 1.2 kcal/mol·nm). This progression involved overcoming a small barrier of 1 kcal/mol representing the opening of the F265 gate, followed by a second saddle representing the opening of the F263 gate. Movement past the third saddle into the N-barrel, which was the narrowest region of the tunnel, was facilitated by gliding hydrophobic interactions between the TG and the N-PL acyl tail extremities. This mechanism resulted in an accelerated movement that prevented kinetic trapping, as local fluctuations of the PL tails tugged TG forward (Fig. 5B). In contrast, the absence of PLs generated a much larger energetic barrier of +2.5 kcal/mol for the transition from the neck to N-barrel (Fig. 5A).

As TG approached the exit of the N-barrel, it interacts with another hydrophobic cluster comprising Y40, Y57, L59, I117, V178, W162, and F167. In this region, the energy barrier sharply rose by 4 kcal/mol. When compared to the deepest point of the PMF at the C-barrel/neck interface, the total barrier reached 5 kcal/mol, which was sufficient to strongly resist spontaneous passage. This steep ascent was further exacerbated as TG moved from the favorable hydrophobic milieu back into an aqueous environment (Fig. 5B). While a similar ascent of approximately +2 kcal/mol occurred in the absence of PL plugs, the free energy value became positive, suggesting native resistance for TG to exit the tunnel without external facilitation (Fig. 5A). A similar result was obtained for 2 alternative paths in the presence or absence of PLs (Figs. S6 and S7).

## Discussion

This study establishes that PL plugs are indispensable modulators of both the structural and functional dynamics of CETP. This is evidenced by 4 mechanistic observations: PL plugs (a) seal the 2 central orifices of the tunnel preventing premature loss of substrate, (b) synchronize barrel domain dynamics, (c) facilitate lipid transit through a gliding mechanism, and (d) are themselves regulated by electrostatic interactions at the headgroups. Based on these findings, we propose a multistep mechanism of lipid transfer (Fig. 5C): (a) PL-induced domain synchrony enables lipoprotein docking and penetration; (b) neutral lipids from donor lipoprotein enter the tunnel via CETP termini, guided by concentration gradients; (c) lipids traverse the tunnel through sequential hydrophobic interactions—the local fluctuations of PL acyl tails provide a deformable environment that buffers lipid movement, gently tugging the lipid forward into N-barrel; (d) Phe residues at the neck function act as a gating system for preventing backflow; (e) hydrophobic interactions with PL acyl chains generate a gliding effect, propelling lipids forward into the N-barrel (the narrowest region of the tunnel); and (f) lipids exit through the distal end, into the recipient lipoprotein. The PMF showed a very high exit barrier, into the aqueous environment, which is strong enough to resist spontaneous diffusive movement of TG. However, the presence of lipoproteins at the ends of CETP may substantially reduce this barrier supporting the formation of ternary CETP complex during lipid transfer.

The physical occlusion of central pores of CETP by PLs enhances the tunnel hydrophobicity. This is corroborated by our thermodynamic analysis, which shows that TG entry into the PL-bound tunnel is energetically more favorable (exhibiting a more negative free energy) than entry into apo-CETP. Beyond sealing, the acyl tails of the PLs actively reroute TG toward the hydrophobic patch at the roof of the neck, directing it onto the productive translocation path. While PLs have previously been proposed to shield lipid cargo from aqueous exposure [17,20,42], their roles in preventing premature exit and rerouting TG have not been demonstrated so far.

Once TG is directed onto the productive path, the PL acyl tails provide a second, more active contribution: They form a hydrophobic planar surface, against which TG glides, producing spikes in instantaneous velocity as it passes through the neck. This gliding mechanism lowers the energetic barrier for the neck-to-N-barrel transition from +2.5 kcal/mol (apo-CETP) to approximately +1.2 kcal/mol (PL-bound), preventing kinetic trapping. Consistent with this observation, PL acyl tails convert CE from a bent to a linear conformation as it traverses the neck [28]; our findings suggest that this linearized CE would similarly glide over the hydrophobic plane, extending the mechanism to both lipid species transported by CETP.

Beyond the role of PLs, our study identifies 3 Phe residues (F263, F265, and F270) that form flaps at the tunnel’s neck region. Given their bottleneck location and distinct open and closed states observed in our simulation, we propose these residues function as directional valves rather than simple hinged gates. In a purely diffusion-driven system, such valves are essential to prevent substrate reflux, especially within a ternary complex, ensuring that once a lipid enters the tunnel, its forward trajectory is maintained. The biological importance of this gating system is underscored by their conservation across BPI/LBP family. The fact that these same residues are energy barriers for both TG and CE [25–27] and that small-molecule inhibitors acting at F263 and neighboring residues block both lipid species [15,26] reinforces the view that this gating system is central to CETP function rather than being incidental. Naturally occurring missense variants along this shared pathway, including I205F, L206R, and C13Y (Table S7), further support this conclusion. The reduced expression of conservative Tyr substitutions indicates that these residues also contribute to structural integrity, and future studies involving thermal shift and circular dichroism using purified proteins will be required to decouple their folding and gating roles.

Beyond sealing the tunnel, PLs synchronize the conformational movements of the N- and

and C-terminal β-barrels. These concerted movements, characterized by squeezing (barrels moving toward each other) and stretching (barrels moving apart), may generate peristaltic forces that propel lipids through the tunnel. This domain synchrony likely facilitates simultaneous binding of 2 lipoproteins. Indeed, CETP’s affinity for second lipoprotein increases after engaging the first, suggesting allosteric communication between the termini [24]. While single-molecule fluorescence resonance energy transfer may be needed to further validate this hypothesis, biochemical evidence suggests that this allostery is markedly reduced by inhibitors such as anacetrapib and torcetrapib, which displace the PL located at the N-barrel–neck interface [15,24]. Consequently, we propose that a key component of the inhibitory mechanism is the loss of PL-mediated domain synchrony, which supplements the direct steric blocking of the tunnel.

Electrostatic interactions surrounding the PL headgroups also play a vital role in modulating CETP stability and activity. Our results show that salt-bridge-forming residues, such as R201 and R424, regulate CETP activity by influencing PL kinetics and tunnel hydrophobicity. Specifically, these interactions enhance favorable hydrophobic contacts with TG, increasing the “gliding” events that propel the lipid through the tunnel. The clinical relevance of this region is underscored by missense variants, such as R282C and D442G, which are associated with hyperalphalipoproteinemia [43]. Furthermore, D442G results in 25% reduction in TG transfer [44], whereas R201 is involved in torcetrapib binding [15]. The solvent-exposed, chemically tractable nature of these pockets makes them attractive targets for next-generation CETP therapeutics that operate via a mechanism distinct from direct tunnel occlusion. However, structure-based design studies will be required to assess their full tractability.

Given their indispensable functional role, CETP likely circulates in plasma in a constitutively PL-bound form. We posit that PL incorporation postsynthesis via lipoprotein binding is unlikely for several structural reasons. First, electron microscopy indicates that the PL-binding pocket is situated >15 Å from the HDL surface upon docking [11,12], creating a physical gap. Second, PL entry through the hydrophobic tunnel followed by a complex internal rotation to seal the orifices is geometrically implausible. Finally, while it has been suggested that CETP might acquire PLs through its central concave groove during lipoprotein binding [17], this scenario fails to account for the highly precise orientation required to effectively plug the tunnel. The conservation of PL orientation across CETP and BPI structures (PDB IDs: 2OBD, 4EWS, and 1BP1) [17,21] strongly suggests cotranslational incorporation. Future folding simulations will be required to confirm this hypothesis. Moreover, if the orientation of PL deviates substantially from that of the crystallographic model, quantitative aspects of the gliding mechanism deduced in this study would require reevaluation.

Despite the mechanistic insights provided here, this study has certain limitations. First, we used DOPC and a single TG species as representatives. CETP likely encounters a diverse lipidome *in vivo*; therefore, variation in acyl chains and unsaturation may quantitatively alter kinetics, but the fundamental hydrophobic effect extended by these lipids to different TG species may remain qualitatively conserved. Second, if the orientation of PL substantially differs from that of the crystal structure, the quantitative aspects of the gliding mechanism would require reevaluation. Similarly, a natural extension of this work would involve direct removal or replacement of PLs to elucidate its effect on transfer activities of TG and CE to further validate the central hypothesis. Third, the reduced expression of Phe-valve mutants suggests that these residues are critical for structural integrity; however, without thermal shift assays or circular dichroism, we cannot definitively decouple misfolding or trafficking defects from gating failure. Finally, our mechanistic model captures the intrinsic tunnel dynamics of CETP and not the full physiological lipid transfer process. It deliberately excludes lipoprotein surfaces to allow for mechanistic dissection of tunnel physics without the confounding complexity of membrane–protein interfaces. Further, it does not elucidate if a CE or TG or 2 TGs/CEs can simultaneously traverse or if the exchange is sequential. Given the tunnel geometry, particularly at the neck region, in which PL plugs reside, and Phe flaps create the narrowest constriction, we speculate that simultaneous passage of 2 TGs may be sterically constrained; however, their tandem-sequential passage may be conceivable. In contrast, simultaneous movement of 2 flexible CEs or a CE and TG may be relatively highly favorable. A full ternary-complex simulation is a necessary and an important next step to understand the dynamics of CETP-dependent lipid transfer.

## Conclusion

Our work provides compelling evidence in favor of the tunnel mechanism of lipid transfer. In summary, we demonstrate that PL plugs act as structural cofactors that enhance the lipid transport efficiency of CETP by modulating tunnel hydrophobicity and protein conformational dynamics. This PL-mediated mechanism may extend to other BPI/LBP family proteins, such as PLTP, LBP, and BPI. F265, F263, and F270 flap residues and salt bridge forming residues are important regulatory points in the CETP structure that influence its stability and activity. While CETP inhibition has focused on tunnel occlusion, requiring hydrophobic drug molecules that can enter the CETP tunnel, design of therapeutics that target the solvent accessible PL-binding pockets may prove to be an exciting alternative.

## Data Availability

The data including simulation parameter files and other input files for classical MD, SMD, and umbrella sampling along with Python scripts used for analysis are available at https://doi.org/10.5281/zenodo.18708665.
