# Supplementary material for: Phospholipids that Plug the Pores of Cholesteryl Ester Transfer Protein Control Its Triglyceride Transfer
Source: Comput Struct Biotechnol J. 2026 Jun 1;35(1):0122. doi: 10.34133/csbj.0122 (PMC13223356; doi:10.34133/csbj.0122)
Supplement: Supplementary 1 — Supplementary Methods Figs. S1 to S7 Tables S1 to S7 Movies S1 to S3 [file csbj.0122.f1.zip › Revision_Supplemental_Info_CSBJ_Rev1.docx]

**Supplementary Information**

**Phospholipids that plug the pores of cholesteryl ester transfer protein control its lipid transfer dynamics**

Sukriti Sacher^a^, Praveen Singh^b,e^, Abhishek Mukherjee^c^, Akash Kumar Bhaskar^d,e^, Kausik Chakraborty^e^, Laurent Counillon^f^, Shantanu Sengupta^e*^, Mallorie Poet^f*^ & Arjun Ray^a*^

^a^Department of Computational Biology, Indraprastha Institute of Information Technology, New Delhi, India -110020

^b^Buck Institute for Research on Aging, Novato, CA, USA -94945

^c^Ambinova Technologies Pvt Ltd, New Delhi, India -110039

^d^Division of Pharmaceutical Sciences, St. Jude Children’s Research Hospital, Memphis, TN, USA - 38105

^e^Institute of Genomics and Integrative Biology, Sukhdev Vihar, Mathura Road, New Delhi, India -110025

^f^Université Côte d’Azur, CNRS, Laboratoire de PhysioMédecine Moléculaire (LP2M), Laboratories of Excellence Ion Channel Science and Therapeutics, Nice, France -06107

**Corresponding author**

**Arjun Ray:** [arjun@iiitd.ac.in](mailto:arjun@iiitd.ac.in)

**Mallorie Poet:** [mallorie.poet@univ-cotedazur.fr](mailto:mallorie.poet@univ-cotedazur.fr)

**Shantanu Sengupta:** [shantanus@igib.res.in](mailto:shantanus@igib.res.in)

**This PDF file includes:**

Supplementary Method

Figs. S1 to S5

Tables S1 to S10

SI References

1. **Supplementary Methods**

**1.2. Calculation of tunnel volume and hydrophobicity**

Simulation frames every 1 ns post-stabilization were extracted using gmx trjconv and were aligned with respect to the last frame. Tunnel volume and hydrophobicity were calculated using CICLOP [1]. Tunnel hydrophobicity is calculated based on side chains of residues lining the tunnel.

**1.3. Bent angle and normal model analysis of CETP**

Normal mode analysis allows for determination of large amplitude motions and vibrational modes exhibited by the protein molecule. It can also determine conformational states that are accessible to a molecule about an equilibrium position [2]. The average bent angle between the two barrels was calculated using gmx angle. To deduce the dominant internal modes of motion, the overall rotation and translation of CETP was removed by least square fitting of each frame of the trajectory to the last structure of simulation. The covariance matrix of atomic coordinates across the trajectory was built and diagonalized using gmx covar. The two principal components were obtained using gmx anaeig. The eigenvalues were converted to nmd using Prody [3,4] and were visualized using normal mode wizard in VMD [5].

**1.4. Phasic movement of CETP barrels**

The angle between the C-barrel and N-barrel domains with respect to the neck region was calculated using gmx angle. The Augmented Dickey Fuller (ADF) test was performed on this time series output to check for its stationarity. A time series is considered stationary if its statistical properties are independent of time. The ADF test assumes that a time series is not stationary owing to the presence of unit root (Null hypothesis) or alternatively, the time series is stationary owing to the absence of unit root (Alternative hypothesis).

Subsequently, a 1-ns moving average was calculated for movements of the C-barrel and N-barrel. The cross-correlation function was calculated as a measure of similarity or coherence between the movement of two barrels with respect to each other. To obtain the minimum-phase response and represent the frequency of this time series as the rate of phase change, the Hilbert transformation was performed as follows:

The analytical signal x_a (t) of signal x (t) is:

*x_a_ = F^-1^(F(x)2U) = x + iy*, (Eq. 1.4.1)

where F is the Fourier transform; U is the unit step function; and y is the Hilbert transform of x.

Hilbert transformation transforms all the components of a signal by 90°, turning a real-valued signal into a complex signal, and allows the assessment of change in phase of a signal. Hilbert transformation of this time series was analyzed by zeroing out the negative half of the frequency spectrum, turning this real-valued signal into a complex signal. The phase angle for movements of the C-barrel and N-barrel at each time point was calculated from the Hilbert-transformed signal. The number of frames, in which the phase angles of the two barrels laid in the same phase (overlapped) or opposite phases (no overlapping) was recorded.

**1.6. Path traversed by TG through the CETP tunnel and residues that make contact**

For each SMD run in the absence or presence of PLs, the position of the C1 carbon of TG was traced throughout the simulation trajectory. The structures were visualized and rendered using UCSF Chimera [6] for tracing TG traversal. Contact analysis was performed using CONAN [7] for identifying CETP residues within the radius of 0.3–0.6 nm of TG as it traversed the tunnel. Residues within this contact radius for > 1 ns were extracted for each SMD run. Residues that were either common in all six runs or found in at least four runs were reported.

**1.7. TG fall out analysis**

A simulation frame from the previously carried out SMD simulations was extracted such that TG had entered the tunnel from the C-barrel mouth and reached its end (end of Phase 1). Starting with this structure, random seeds in the presence and absence of PL plugs were set to generate random velocities assigned from Maxwell-Boltzmann distribution, and TG was pulled towards the neck (43 simulations) (Phase 2). For each replicate, the distance between the center of mass of TG and an arbitrary vector passing through the CETP tunnel was calculated and plotted to show the path taken by TG through the neck.

**1.8. Power-stroke like movement of PL acyl tails**

The distance traversed by TG (center of mass of the glycerol headgroup) and center of mass of residues in the N-barrel region was calculated using gmx distance. The instantaneous velocity, defined as the rate of change in velocity for a very small-time interval (Δt = 10 ps), was calculated for simulations in the presence or absence of PLs. These values were denoised based on the distribution of instantaneous velocities obtained throughout the SMD replicates.

**1.9. Side-chain movement of Phe residues lining the neck**

For the three Phe residues lining the neck, the torsion angle of 𝝌_2_ side chain was calculated using dihedral module of the MD Analysis suite [8, 9]. The side chains frequently undergo switching between rotamers; however, we were interested in deviation of the side chain angle irrespective of the rotameric states. Two rotameric states were identified by clustering the torsion angle of 𝝌_2_ side chain throughout simulation. Rotamericity of Phe residues substantially varies depending on their positions in a protein [10]. Therefore, the mean of the two rotamers exhibited by the Phe residues was separately considered (Supplementary Fig. S3A,B). These means were subtracted from the respective values across time. The deviation in change of 𝝌_2_ irrespective of the rotamer switch was then calculated.

**1.10. Salt bridge analysis**

Salt bridges between the PL headgroup and charged amino acids surrounding the ventral openings (Lys, Arg, Glu, and Asp) were analyzed using an in-house python script. Salt-bridge distances were calculated between basic nitrogen atoms and acidic oxygen atoms in the PL headgroup or surrounding amino acids. A salt bridge was said to exist if the distance between the two groups was < 6 Å [11,12].

**1.11. Fluctuation and stability of the hydrophobic plane**

Root mean square fluctuation of the PL heavy atoms was calculated while TG moved through the tunnel, using gmx rmsf. The plane formed by PL acyl tails was measured by calculating the vectors formed between C29 and C218 (vector1), and between C39 and C318 (vector2). The angle between the two vectors depicts the overall conformation of PL tails in the tunnel, such that an angle close to 0° represents that the two tails of PL are parallel to each other.

1. **Supplementary Figures**


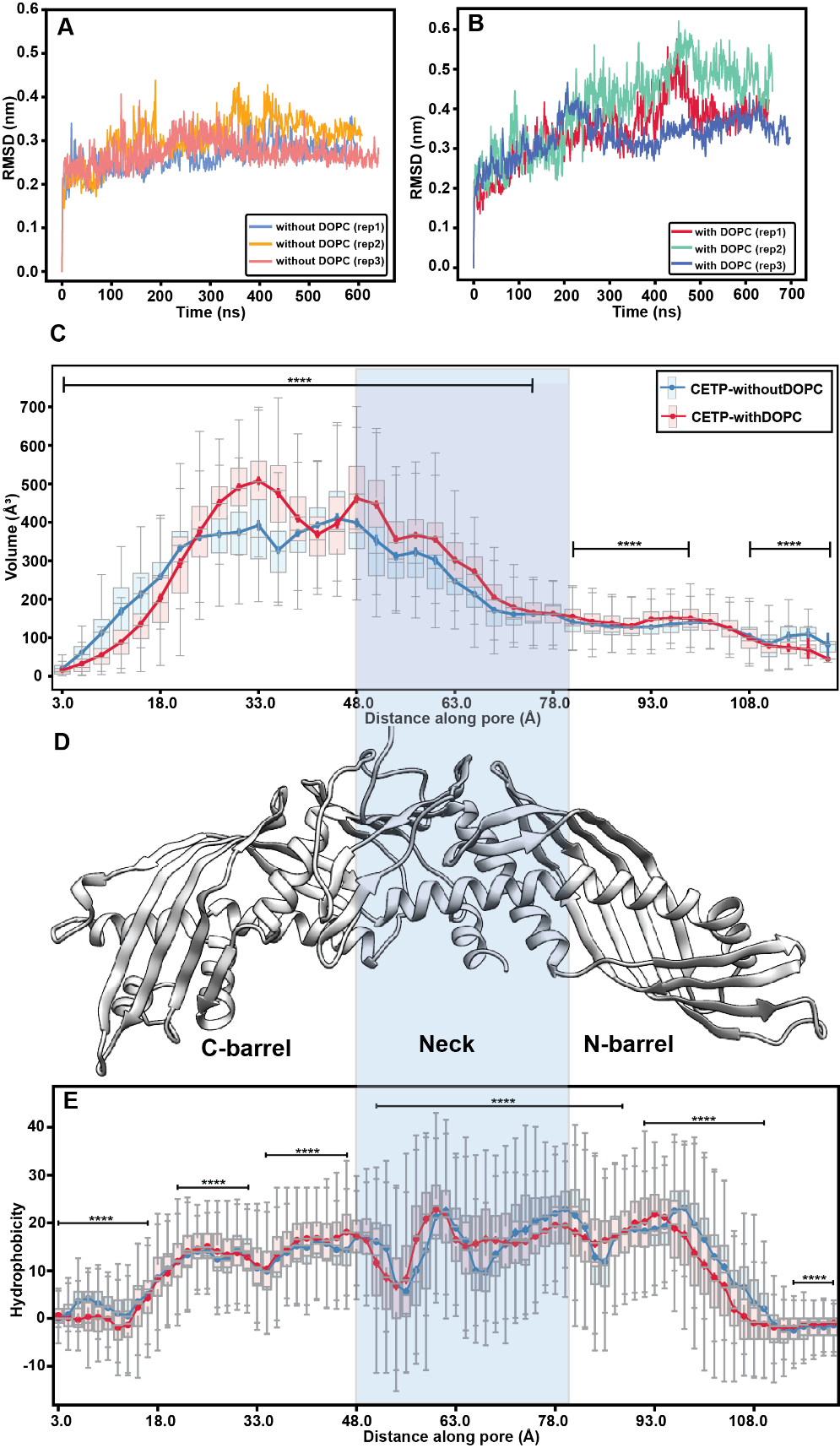


**Figure S1** Root mean square deviation of the backbone atoms of CETP **(A)** without PL plugs and **(B)** with PL plugs. **(C)** Volume profile of CETP in presence and absence of PL plugs. Simulation frames post stabilization were aligned such that the CETP tunnel aligned along the z-axis. These structures were used to calculate volume and hydrophobicity using CICLOP. **(D)** Structure of CETP showing the three domains. **(E)** Hydrophobicity profile of CETP in presence and absence of PL plugs. The box plot depicts the distribution of volume and hydrophobicity values calculated over 150 frames (post stabilization). Solid lines depict the median value of the distribution for the distance along the tunnel axis. Mann–Whitney U test was used to calculate the statistical significance of the difference between the two profiles (**p* < 0.05, ***p* < 0.01, ****p* < 0.001, *****p* < 0.0001).


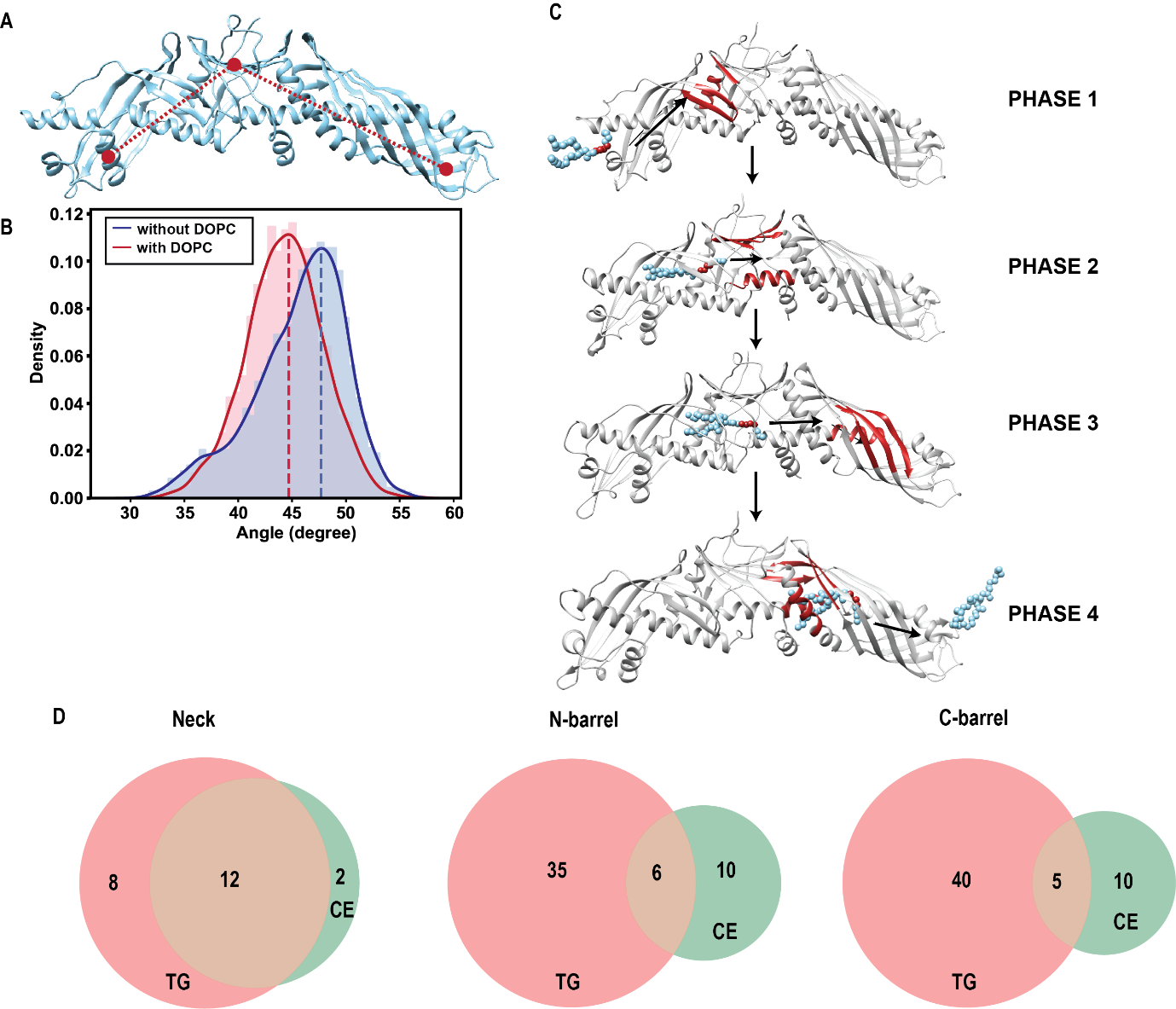


**Figure S2 (A)** The vectors used to define the average bend angle of CETP. **(B)** Average bend angle of the two barrels with respect to the neck of CETP. **(C)** Schematic depicting the four phases in which TG was steered through the CETP tunnel in absence or presence of PL plugs. TG was placed at the entrance of the C-barrel domain. A pull force was applied on the center of mass of the termini atoms of TG (highlighted in red) to pull it towards the center of mass of CETP tunnel residues in the direction of the N-terminal barrel (red). Briefly, edge of C-barrel (Phase 1), edge of neck (Phase 2), edge of N-barrel (Phase 3) and subsequently out of CETP through the N-barrel end (Phase 4) by applying a push force instead of pull force. **(D)** Comparison of residues that TG and CE interact with through their journey through the CETP tunnel.


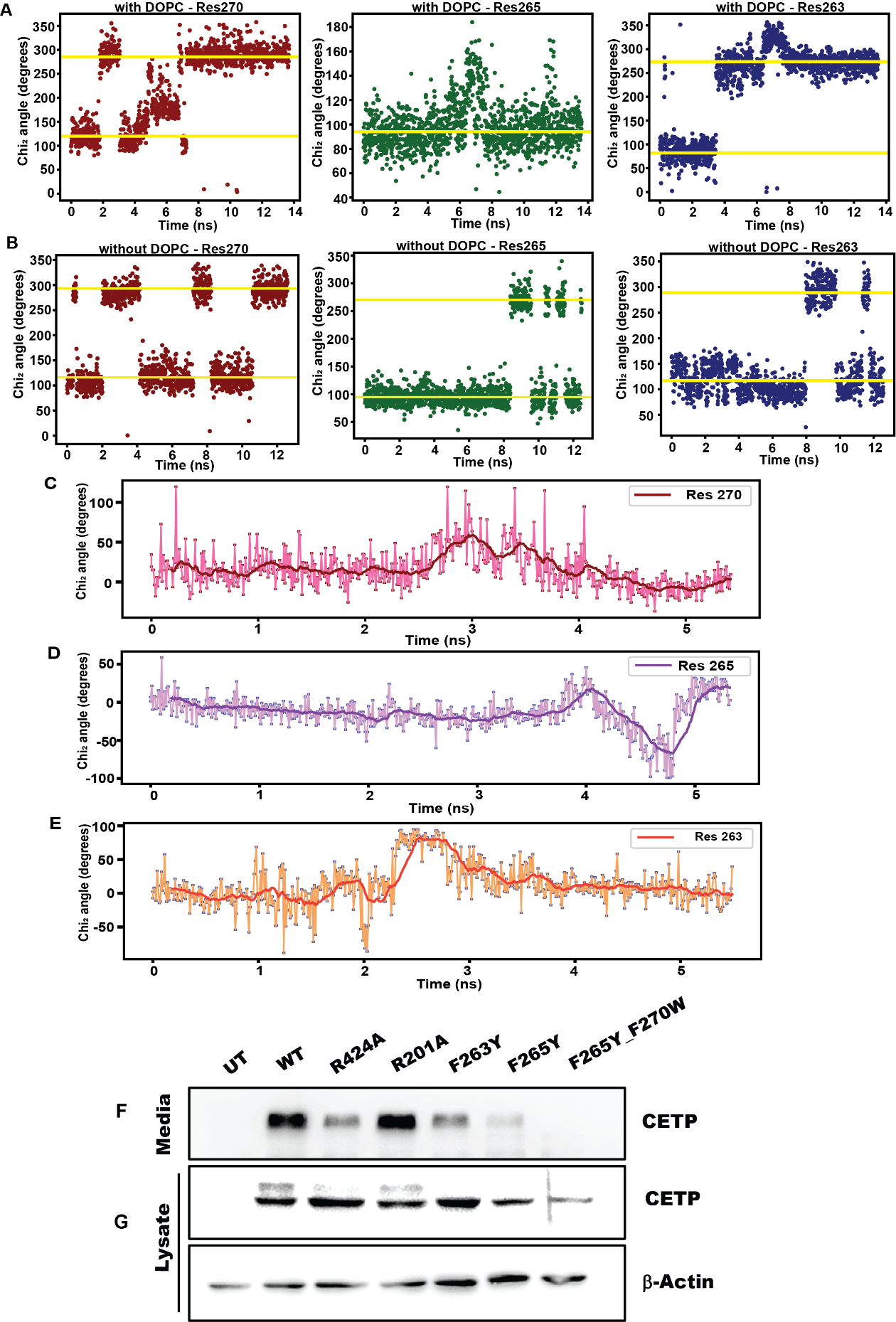


**Figure S3** The average of the cluster of for 𝝌_2_ angle of Phe from simulations in **(A)** presence and **(B)** absence of PL were calculated and was taken as the rotameric form. These averages are depicted with a yellow line. In the presence of PL, the two rotameric forms were, F270: 120° and 285°, F265: 94°, F263: 82° and 274°. In the absence of PLs, the two rotameric forms were, F270: 116° and 293°, F265: 95° and 270°, F263: 117° and 289°. While side chains of these residues frequently switched between these rotameric forms, a hinge-like opening motion was only apparent in the presence of PLs. Changes in normalized 𝝌_2_ angle of **(C)** Phe270 **(D)** Phe265 and **(E)** Phe263 throughout the Phase 2 of simulation when TG was pulled from the N-terminus towards C-terminus. Solid line depicts the moving average across 20 ps in a simulation, while line plot represents the changes in 𝝌_2_ angle in real time. Protein expression of CETP in **(F)** media or **(G)** cell lysate analyzed by immunoblotting using anti-V5 antibody. One representative blot is shown. β-Actin is used as internal control for expression in cell lysate.


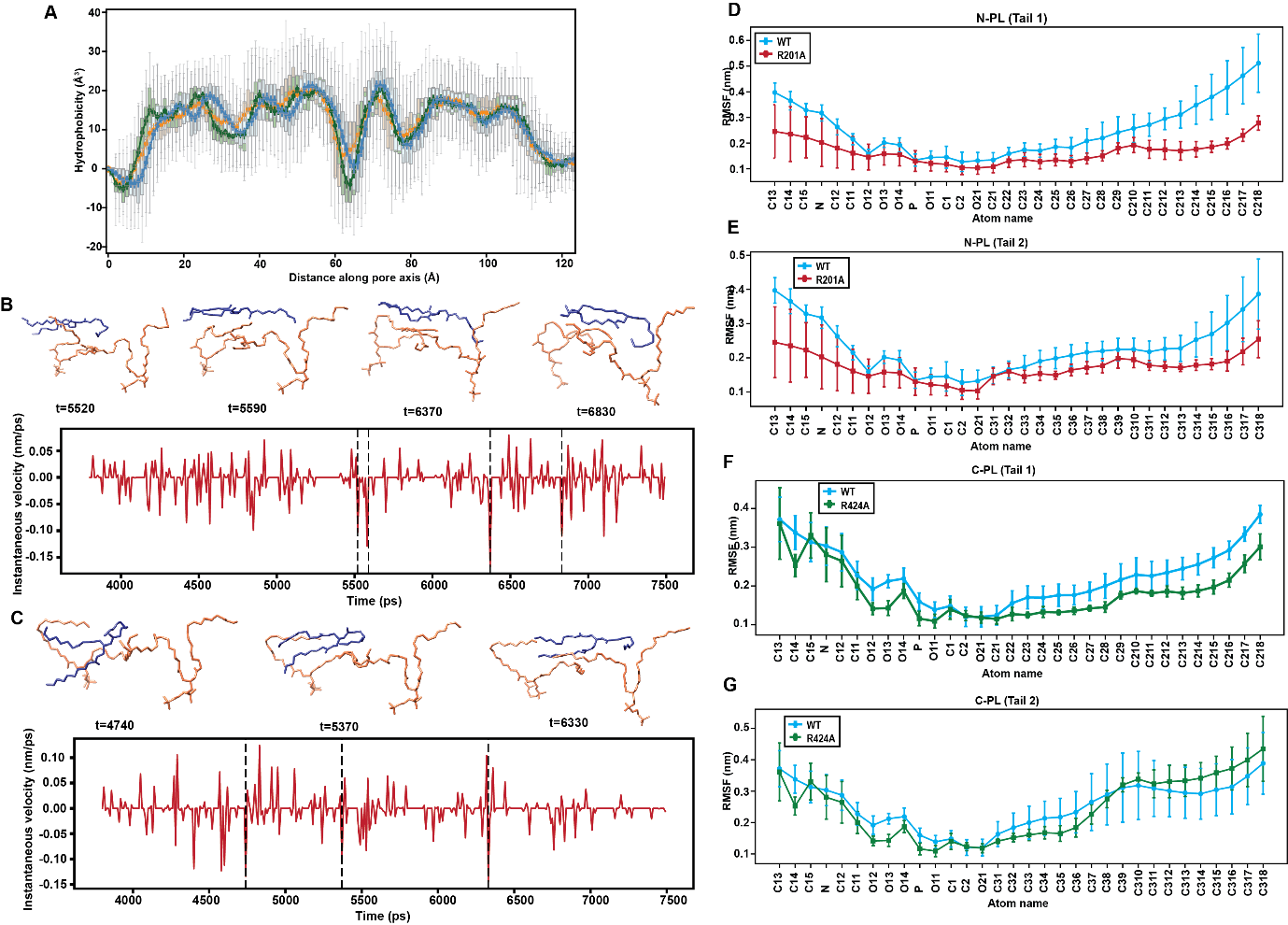


**Figure S4 (A)** Hydrophobicity profile of WT-CETP with respect to mutants. Simulation frames post stabilization were aligned such that the CETP tunnel aligned along the z-axis. These structures were used to calculate hydrophobicity using CICLOP. The box plot depicts the distribution hydrophobicity values calculated over 100 frames. Solid lines depict the median value of the distribution for the distance along the tunnel axis of WT-CETP (blue), R201A orange) and R424A (green). Instantaneous velocity of TG through CETP in the presence of PL plugs. **(B)** and **(C)** represent two separate instances from two independent runs where powerstroke-like-movement was observed. The jumps in instantaneous velocity are highlighted with a dashed line. The position of TG with respect to PL acyl tails is also depicted as snapshots from the simulations. Root mean square fluctuation of the **(D)** and **(E)** N- and **(F)** and **(G)** C-PL during steered molecular dynamic simulations.


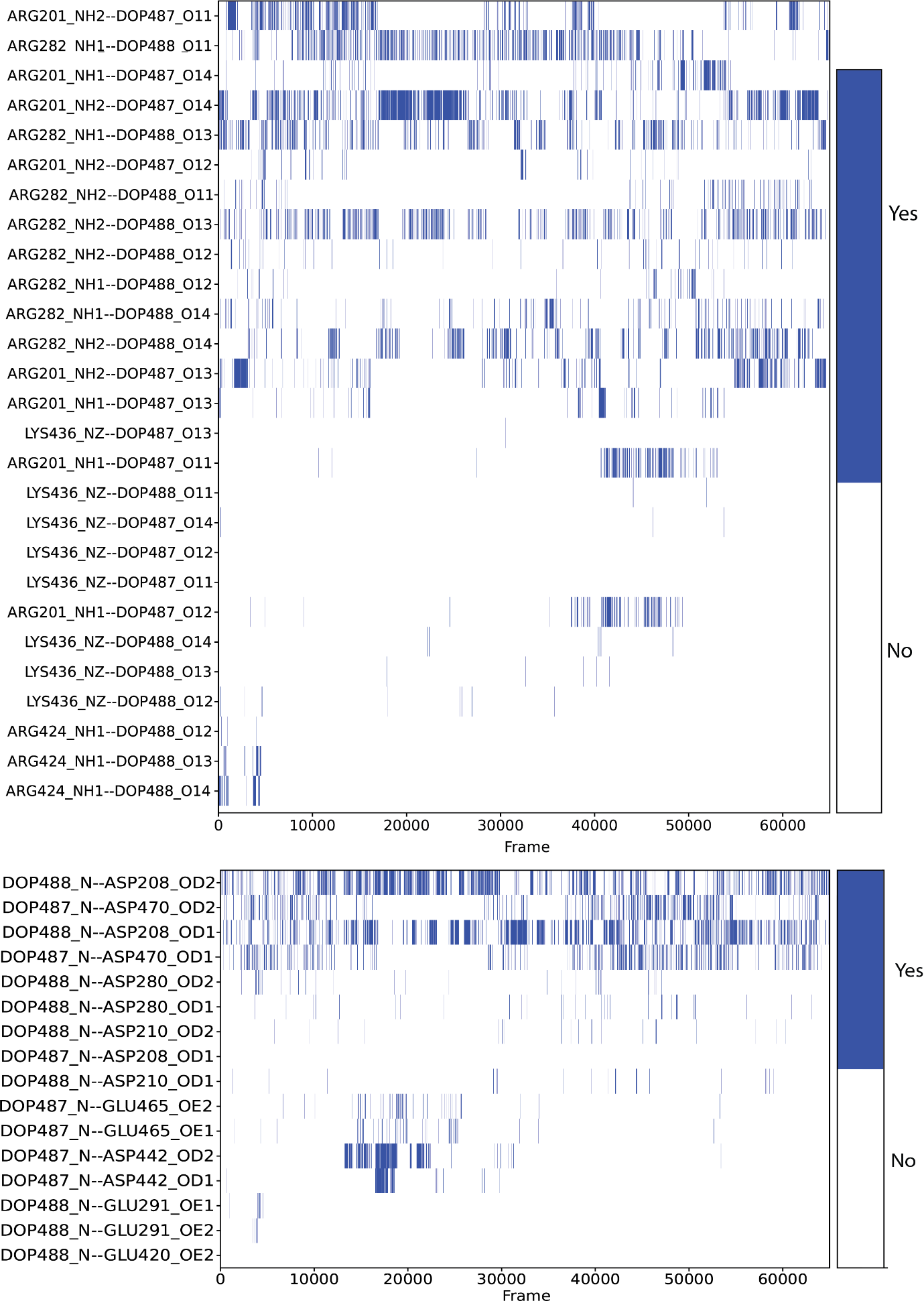


**Figure S5:** Salt bridges between PL headgroup and charged residues lining the PL pockets. Salt bridge distances were computed between the basic nitrogens and acidic oxygens in PL headgroup or amino acids surrounding the PL within 6Å.


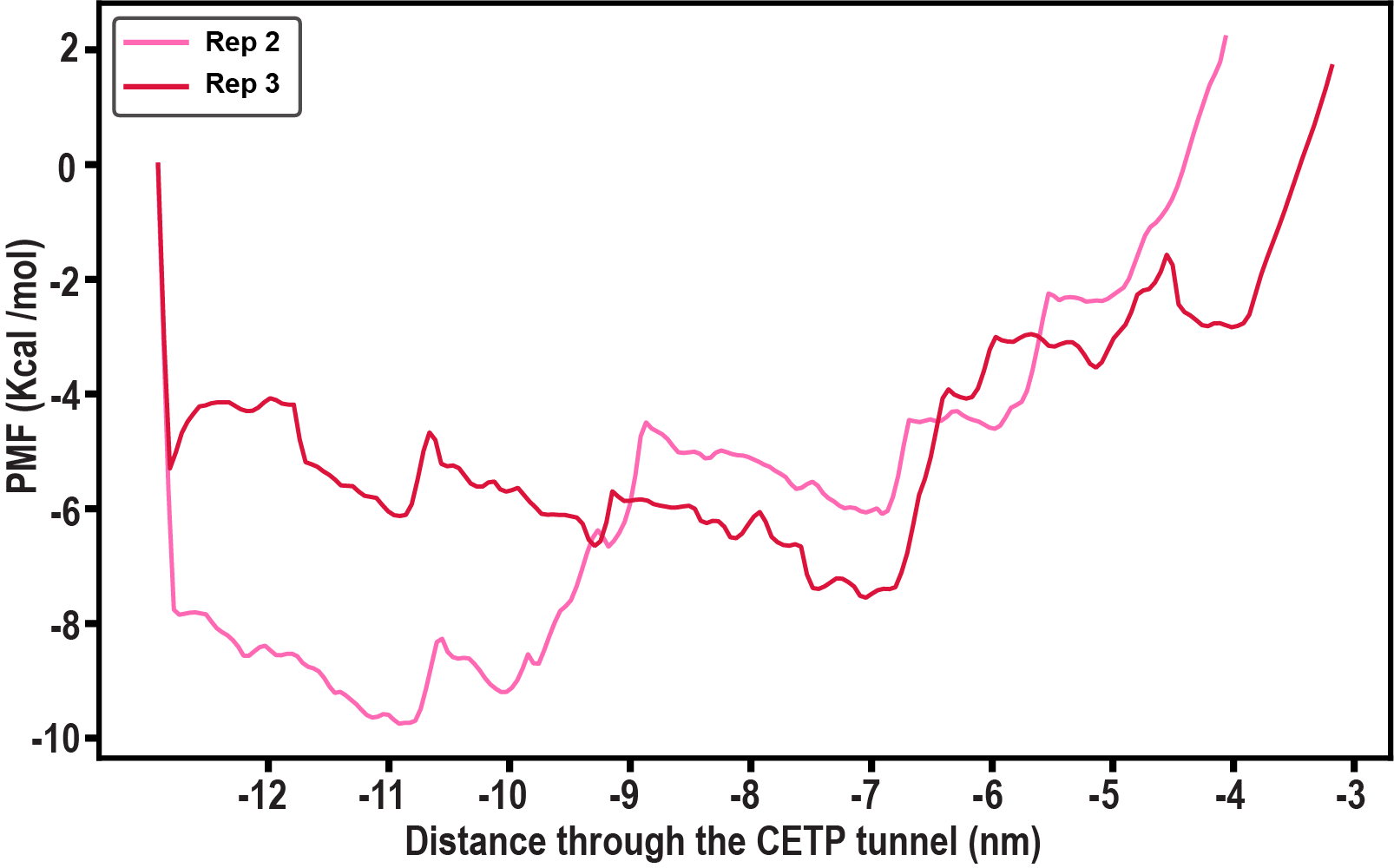


**Figure S6:** Potential of mean force profile for the movement of TG through the CETP tunnel in the presence of PL plugs for two different paths derived from two separate SMD runs.


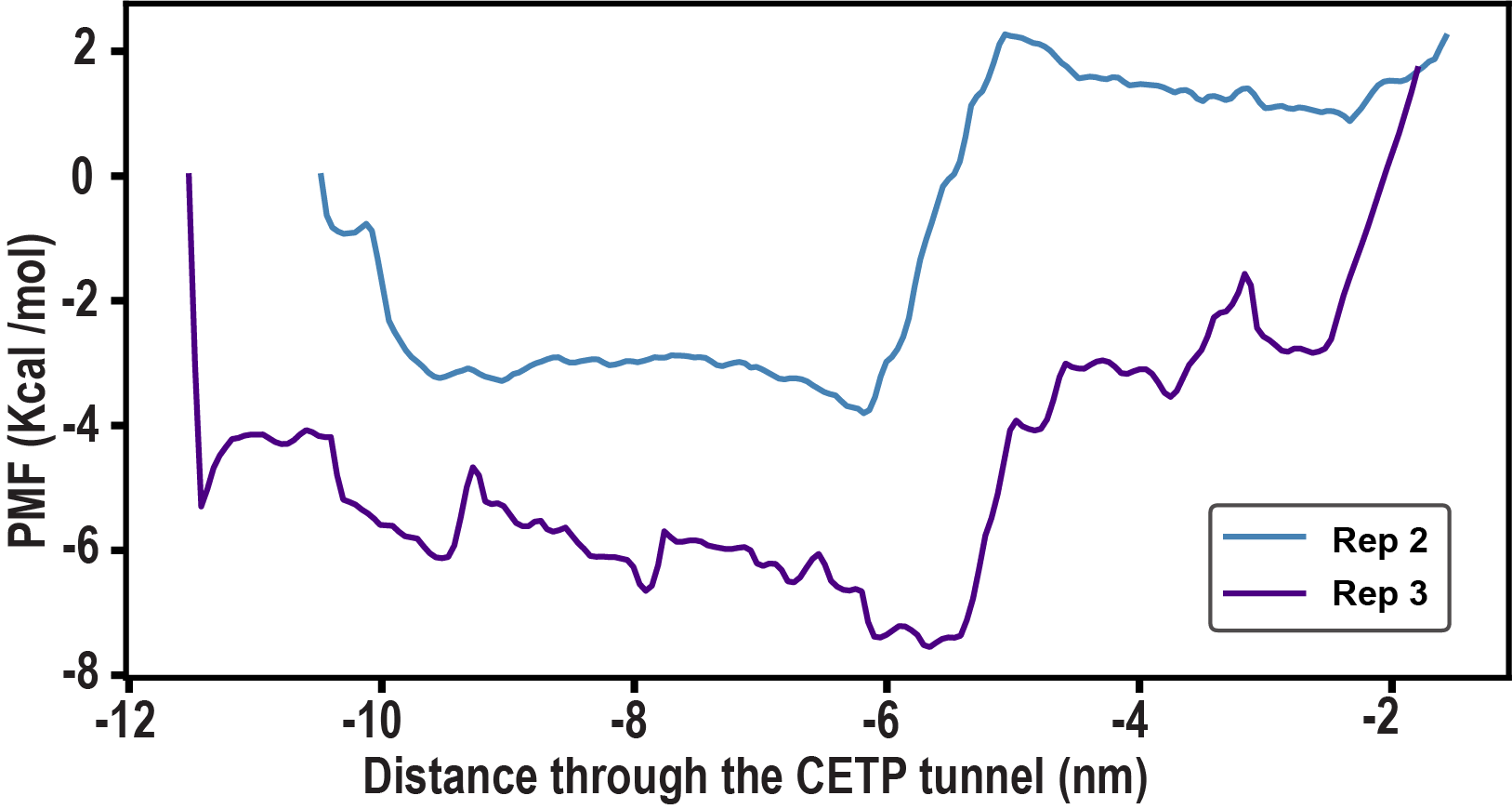


**Figure S7:** Potential of mean force profile for the movement of TG through the CETP tunnel in the presence of PL plugs for two different paths derived from two separate SMD runs.

1. **Supplementary Tables**

**Table S1: Oligonucleotide sequence for verification of mutant CETP plasmids**

| **Name** | **Oligonucleotide sequence 5' — 3'** |
| --- | --- |
| **F263Y_CETP fwd** | CCCCGCATGCTGTACTACTGGTTCTC |
| **F263Y_CETP rev** | GAGAACCAGTAGTACAGCATGCGGG |
| **F265Y_CETP fwd** | GTACTTCTGGTACTCTGAGCGAGTC |
| **F265Y_CETP rev** | GACTCGCTCAGAGTACCAGAAGTAC |
| **F270Y_CETP fwd** | CTCTGAACGGGTCTGGCACTCGCTGGC |
| **F270Y_CETP rev** | GCCAGCGAGTGCCAGACCCGTTCAGAG |
| **R201A_CETP fwd** | GTCCAGACAGCGGCTGCCAGCAT |
| **R201A_CETP rev** | ATGCTGGCAGCCGCTGTCTGGAC |
| **R424A_CETP fwd** | GATTATCACTCGGCATGGCTTCCTG |
| **R424A_CETP rev** | CAGGAAGCCATGCCGAGTGATAATC |
| **seq-fwd1_CETP** | CACCCTGAAGCTGGTCCTGAAGGG |
| **seq-fwd2_CETP** | CCCAGACCAGCAACATTCTGTAGC |

# Table S2. Simulation statistics highlighting the number of successful runs for each phase of the SMD pull.

| System | Phase 1 | Phase 2 | Phase 3 | Phase 4 |
| --- | --- | --- | --- | --- |
| CETP-without DOPC | 7 | 5/7 = 0.71 | 5/5 =1 | 5/5 = 1 |
| CETP-with DOPC | 7 | 7/7 = 1 | 7/7 = 1 | 7/7 = 1 |

#

# Table S3. Residues that make contact with TG > 1ns of simulation time in 66% of replicates of CETP without PL plugs.

| Domain | Residue |
| --- | --- |
| C-barrel | 270, 273, 274, 277, 283, 285, 307, 308, 311, 321, 323, 325, 329, 331, 338, 340, 342, 344, 346, 348, 350, 361, 363, 367, 371, 373, 375, 380, 382, 394, 397, 398, 405, 408, 409, 412, 413, 416, 417, 418, 421, 422, 425, 429, 433 |
| Neck | 15, 195, 198, 202, 205, 206, 228, 236, 261, 263, 265, 438, 439, 441, 448, 455, 457, 459, 467, 476 |
| N-barrel | 20, 23, 24, 27, 28, 31, 35, 64, 67, 69, 74, 82, 84, 86, 89, 91, 93, 117, 119, 121, 123, 125, 127, 145, 147, 150, 152, 167, 172, 175, 176, 179, 180, 183, 184, 187, 190, 194 |

#

# Table S4. Residues that make contact with TG > 1ns of simulation time in 66% of replicates of CETP with PL plugs.

| Domain | Residue |
| --- | --- |
| C-barrel | 269, 270, 273, 283, 285, 287, 292, 295, 296, 310, 311, 321, 323, 331, 338, 340, 344, 346, 348, 350, 352, 359, 361, 363, 367, 369, 371, 373, 380, 382, 405, 406, 408, 409, 412, 413, 416, 417, 418, 420, 421, 422, 425, 429, 432 |
| Neck | 11, 13, 15, 195, 198, 199, 201, 202, 205, 206, 215, 228, 230, 232, 236, 263, 265, 432, 448, 455, 457, |
| N-barrel | 20, 23, 24, 27, 28, 31, 35, 40, 64, 67, 69, 72, 74, 82, 84, 86, 89, 91, 93, 117, 119, 121, 123, 125, 127, 129, 136, 138, 145, 147, 150, 152, 167, 172, 175, 176, 179, 183, 187, 191, 194 |

# Table S5. CETP residues that interact with CE taken from reference 25,26

| Domain | Residue |
| --- | --- |
| C-barrel | **321, 350, 292, 301, 270, 429, 303, 433, 304, 305, 308, 309, 312, 315,**  **318** |
| Neck | **230, 263, 232, 265, 202, 198, 199, 205, 206, 15, 13, 236, 461, 441** |
| N-barrel | **162, 99, 35, 34, 167, 40, 108, 112, 115, 119, 118, 23, 57, 59, 93, 158** |

# Table S6. Simulation statistics depicting the number of successful runs spanning Phase2 of the simulation, in the presence and absence of PL plugs.

| System | Phase 1 | Phase 2 (success) | Phase 2 (failure) |
| --- | --- | --- | --- |
| CETP-without DOPC | 7 | 21/43 = 0.48 | 22/43 = 0.5 |
| CETP-with DOPC | 7 | 43/43 =1 | 0/43 = 0 |

**Table S7. Missense single nucleotide variations of TG interacting residues along with their pathogenicity prediction**

| **Sno.** | **Accession** | **dbSNP ID** | **Protein change** | **Location** | **Mapped on protein** | **Condition(s)** | **SIFT** | **PolyPhen2** | **Panther** |
| --- | --- | --- | --- | --- | --- | --- | --- | --- | --- |
| 1 | VCV003491136 |  | I28V | Neck | I11 | **-** | **-** | possibly damaging | **-** |
| 2 | VCV003143283 | rs2543645399 | V153G | N-barrel | V136 | **-** | **-** | probably damaging | probably benign |
| 3 | VCV003143280 | rs762811493 | I430V, I370V | C-barrel | I413,I353 | **-** | **-** | benign | probably benign |
| 4 | VCV003019020 | rs760836166 | M439I, M379I | C-barrel | M422, M362 | **-** | **-** | benign | probably bengin |
| 5 | VCV002980442 | rs144949752 | R369H, R309H | C-barrel | R352, R292 | **-** | Tolerated | benign | probably bengin |
| 6 | VCV002864541 | rs1323326418 | V252E, V312E | C-barrel | V235, V295 | **-** | **-** | possibly damaging | **-** |
| 7 | VCV002722544 | rs36122917 | Y318C, Y378C | C-barrel | Y301, Y361 | **-** | Tolerated | probably damaging | probably bengin |
| 8 | VCV002623272 | rs1567476542 | V373G, V433G | C-barrel | V356, V416 | **-** | **-** | probably damaging | probably benign |
| 9 | VCV002539456 | rs142459781 | R299C | Salt-bridge | R282 | **-** | Deleterious | **-** | **-** |
| 10 | VCV002302010 | rs2543643841 | I81N | N-barrel | I64 | **-** | **-** | probably damaging | probably benign |
| 11 | VCV002301233 | rs933760754 | I222F | Neck | I205 | **-** | **-** | benign |  |
| 12 | VCV002238319 | rs781348259 | V357G, V297G | C-barrel | V340, V280 | **-** | **-** | probably damaging | probably benign |
| 13 | VCV002173132 | rs147412224 | V388I, V328I | C-barrel | V311, V371 | **-** | Tolerated | benign | probably benign |
| 14 | VCV002078436 | rs5887 | V426M, V486M | C-barrel | V409, V469 | **-** | Deleterious | **-** | probably benign |
| 15 | VCV001804584 | rs369941536 | L290P | C-barrel | L273 | **-** | Deleterious | probably damaging | **-** |
| 16 | VCV001405295 | rs141310739 | V280I, V340I | C-barrel | V263, V323 | Hyperalphalipoproteinemia | Tolerated | benign | probably benign |
| 17 | VCV001366232 | rs200134880 | V361A, V301A | C-barrel | V344, V284 | **-** | Tolerated | probably damaging | probably benign |
| 18 | VCV001357558 | rs1164754201 | L449I, L389I | C-barrel | L432, L372 | Hyperalphalipoproteinemia | **-** | possibly damaging | probably benign |
| 19 | VCV001314022 | rs771585518 | C30Y | Neck | C13 | **-** | **-** | probably damaging | probably benign |
| 20 | VCV001305453 | rs774433762 | R309C, R369C | C-barrel | R292, R352 | **-** | **-** | **-** | probably benign |
| 21 | VCV000886619 | rs747056056 | L223R | Neck | L206 | Hyperalphalipoproteinemia | **-** | possibly damaging | **-** |
| 22 | VCV000885657 | rs1326253991 | D399H, D459H | C-barrel | D382, D442 | Hyperalphalipoproteinemia | **-** | **-** | probably benign |
| 23 | VCV000319993 | rs5880 | A390P, A330P | C-barrel | A373, A313 | Hyperalphalipoproteinemia | Deleterious | probably damaging | probably benign |
| 24 | VCV000319976 | rs371258270 | V106M | N-barrel | V89 | Hyperalphalipoproteinemia | Deleterious | probably damaging | probably benign |
| 25 | VCV003716673 |  | A212D | Neck | A195 | **-** | **-** | possibly damaging | **-** |

1. **Supplementary References**
2. P. Garg, S. Sacher, Mrinal, Atul, P. Gautam, A. Ray, CICLOP: a robust and accurate computational framework for protein inner cavity detection, Bioinform. 38 (2022) 2153 – 2161. 10.1093/bioinformatics/btac061.
3. J.A. Bauer, J. Pavlović, V. Bauerová-Hlinková, Normal Mode Analysis as a Routine Part of a Structural Investigation, Molecules 24 (2019) 3293. 10.3390/molecules24183293.
4. S. Zhang, J.M. Krieger, Y. Zhang, C. Kaya, B. Kaynak, K. Mikulska-Ruminska, P. Doruker, H. Li, I. Bahar, ProDy 2.0: increased scale and scope after 10 years of protein dynamics modelling with Python, Bioinform. 37 (2021) 3657 – 3659. 10.1093/bioinformatics/btab187.
5. A. Bakan, L.M. Meireles, I. Bahar, ProDy: protein dynamics inferred from theory and experiments, Bioinform. 27 (2011) 1575 – 1577. 10.1093/bioinformatics/btr168.
6. W. Humphrey, A. Dalke, K. Schulten, K. VMD: visual molecular dynamics, J. Mol. Graph. 14 (1996) 33 – 38, 27 – 28. 10.1016/0263-7855(96)00018-5.
7. E.F. Pettersen, T.D. Goddard, C.C Huang, G.S. Couch, D.M. Greenblatt, E.C. Meng, T.E. Ferrin, UCSF Chimera—A visualization system for exploratory research and analysis, J. Comput. Chem. 25 (2004) 1605 – 1612. 10.1002/jcc.20084.
8. D. Mercadante, F. Gräter, F. C. Daday, CONAN: A Tool to Decode Dynamical Information from Molecular Interaction Maps., Biophys. J. 114 (2018) 1267 – 1273. 10.1016/j.bpj.2018.01.033.
9. R. J. Gowers, M. Linke, J. Barnoud, T. J. E. Reddy, M. N. Melo, S. L. Seyler, D. L. Dotson, J. Domanski, S. Buchoux, I. M. Kenney, and O. Beckstein, MDAnalysis: A Python package for the rapid analysis of molecular dynamics simulations, in: S. Benthall and S. Rostrup (Eds.), Proceedings of the 15th Python in Science Conference, Austin, Texas, 2016, pp. 98 – 105. doi:10.25080/Majora-629e541a-00e.
10. N. Michaud-Agrawal, E.J. Denning, T.B. Woolf, O. Beckstein, MDAnalysis: a toolkit for the analysis of molecular dynamics simulations, J. Comput. Chem. 32 (2011) 2319 – 2327. 10.1002/jcc.21787.
11. H. Schrauber, F. Eisenhaber, P. Argos, Rotamers: To be or not to be?: An Analysis of Amino Acid Side-chain Conformations in Globular Proteins, J. Mol. Biol. 230 (1993) 592 – 612. 10.1006/jmbi.1993.1172.
12. J. Luo, H. Yang, B-L. Song, Mechanisms and regulation of cholesterol homeostasis, Nat. Rev. Mol. Cell Biol. 21 (2020) 225 – 245. 10.1038/s41580-019-0190-7.
13. O. Beckstein, E.J. Denning, J.R. Perilla, T.B. Woolf, Zipping and Unzipping of Adenylate Kinase: Atomistic Insights into the Ensemble of Open   ↔   Closed Transitions, J. Mol. Biol. 394 (2009) 160 – 176. 10.1016/j.jmb.2009.09.009.
